# *COD::CIF::Parser*: an error-correcting CIF parser for the Perl language

**DOI:** 10.1107/S1600576715022396

**Published:** 2016-02-01

**Authors:** Andrius Merkys, Antanas Vaitkus, Justas Butkus, Mykolas Okulič-Kazarinas, Visvaldas Kairys, Saulius Gražulis

**Affiliations:** a Vilnius University Institute of Biotechnology, Graiciuno 8, LT-02241 Vilnius, Lithuania; b Time.ly Network Inc., 2nd Floor, 607 Front Street, Nelson, British Columbia, Canada V1L 4B6; c Vilnius University Faculty of Mathematics and Informatics, Naugarduko 24, LT-03225 Vilnius, Lithuania

**Keywords:** CIF parsers, Perl, Crystallography Open Database

## Abstract

A syntax-correcting CIF parser, *COD::CIF::Parser*, is presented that can parse CIF 1.1 files and accurately report the position and the nature of the discovered syntactic problems. In addition, the parser is able to automatically fix the most common and the most obvious syntactic deficiencies of the input files. Bindings for Perl, C and Python programming environments are available. Based on *COD::CIF::Parser*, the *cod-tools* package for manipulating the CIFs in the Crystallography Open Database (COD) has been developed. The *cod-tools* package has been successfully used for continuous updates of the data in the automated COD data deposition pipeline, and to check the validity of COD data against the IUCr data validation guidelines. The performance, capabilities and applications of different parsers are compared.

## Abstract 2

A syntax-correcting CIF parser, *COD::CIF::Parser*, is described that can parse CIF 1.1 files and accurately report the position and nature of the discovered syntactic problems while automatically correcting the most common and the most obvious syntactic deficiencies.

## 1. Introduction   

Over the quarter century of its existence, the Crystallographic Information Framework (CIF, also Crystallographic Information File; Hall *et al.*, 1991 ▸) – a standard format for reporting and storing data pertaining to crystal structures – has been widely adopted as a standard for supplementary material by the International Union of Crystallography (IUCr) (Brown & McMahon, 2002 ▸) and has been used by the majority of crystallographic journals as well as structural databases [Inorganic Crystal Structure Database (http://www2.fiz-karlsruhe.de/icsd_home.html; Belsky *et al.*, 2002 ▸), Cambridge Structural Database (http://www.ccdc.cam.ac.uk/products/csd/; Groom & Allen, 2014 ▸), CRYSTMET (http://www.tothcanada.com/databases.htm; Le Page & Rodgers, 2005 ▸) and Crystallography Open Database (COD; http://www.crystallography.net/; Gražulis *et al.*, 2012 ▸)]. New CIF dictionaries have been developed with the aim of unambiguously defining ontologies in order to uniformly present data in various fields of crystallography, with notable examples including macromolecular crystallography (Fitzgerald *et al.*, 2006 ▸), powder diffraction (Toby *et al.*, 2003 ▸) and electron density studies (Mallinson & Brown, 2006 ▸). We assume that the main reasons for the CIF format’s popularity are the use of human-readable text, a relatively simple syntax, extensibility, a continued support by the IUCr and an increasing availability of software for CIF processing.

A wide variety of software tools have been developed for reading, writing, validating, manipulating and visualizing CIFs (McMahon, 2006*a* ▸). The unprecedented development in the field of *in silico* simulation of materials sparked the emergence of high-level libraries for materials analysis, such as *AiiDA* (Pizzi *et al.*, 2016 ▸), *ASE* (Bahn & Jacobsen, 2002 ▸) and *pymatgen* (Ong *et al.*, 2013 ▸), which support structural data input in the CIF format. Obviously, the ability to correctly convert CIF into internal data structures (parsing) is of great importance to all of the aforementioned software tools. Examples of general purpose CIF parsers include *vcif* (http://www.iucr.org/resources/cif/software/archived/vcif-1.2; Mc­Mahon, 2006*b* ▸) and *vcif2* (also known by the name of the executable file *cif2cbf*; Todorov & Bernstein, 2008 ▸) in C, *ucif* (Gildea *et al.*, 2011 ▸) in C++, *cif2cif* (Hall & Bernstein, 1996 ▸) in Fortran and *PyCIFRW* (Hester, 2006 ▸) in Python (van Rossum, 2003 ▸). Another noteworthy tool is the *ZINC* package (Stampf, 2004 ▸), which provides a set of converters from CIF to ZINC format and allows convenient manipulation of data in a command line environment. Finally, since the syntax of CIF 1.1 is a subset of the more general STAR 1 (Hall & Spadaccini, 1994 ▸) format, STAR parsers like *STAR::Parser* (Bluhm, 2000 ▸) in Perl (Wall *et al.*, 2000 ▸) and *StarTools* (Keller, 2013 ▸) in Java are also capable of parsing CIFs.

The described parsers are more than adequate when dealing with syntactically correct CIFs. However, some discrepancies occasionally occur between the CIF standard and the supplementary files of published articles. Such files cause problems in automatic processing, for instance when they are ingested by the COD. Minor deviations from the CIF syntax (missing quotes, missing data block headers, duplicated data names *etc*.) appeared to be relatively common and too numerous to be fixed by human editors. It was deemed too inefficient to demand CIF editors (who are often volunteers working in their spare time) to remedy such technical discrepancies between the uploaded text and the CIF syntax. Moreover, when a syntax error is detected by a CIF parser, whether correctable or not, it is extremely important to locate that error as precisely as possible, and to formulate a human- and machine-readable error message that would direct further processing of the input file.

For these reasons we have created a syntax-correcting CIF parser, *COD::CIF::Parser*, that can parse CIF 1.1 files and accurately report the position and nature of the discovered syntactic problems. In addition, the parser is able to automatically fix the most common and the most obvious syntactic deficiencies. Based on *COD::CIF::Parser* we have developed *cod-tools* 1 – a set of tools for manipulating the CIFs in the COD. The* cod-tools* package has been successfully used for continuous updates of the data in the automated COD data deposition pipeline and to check the validity of the COD data against the IUCr data validation guidelines (ftp://ftp.iucr.org/pub/dvntests).

## 2. Methods   

### 2.1. Programming tools   

To present a high-level user interface while maintaining portability, *COD::CIF::Parser* was implemented in Perl. We find the Perl programming language to be ideal for CIF data processing tasks: it has a rich set of text processing operators and native support for enhanced regular expressions, permitting concise formulation of algorithms (Wall *et al.*, 2000 ▸). The long history of consistent Perl development allowed us to gain experience in using this language; the robust and fast compiler gives good performance when compared to other interpreted languages. There are a large number of Perl libraries in CPAN (http://www.cpan.org/) to supplement our own developments. Thus Perl was considered a suitable language to develop the first CIF parser prototype, and it continues to serve as a facile test-bed for future developments.

The main and probably the only drawback of the Perl-implemented parser is its relatively low speed. A noticeably faster but also portable parser can be written in lower-level languages, C in particular. Maintaining C code, however, is significantly more labour intensive than maintaining Perl code. Therefore we re-implemented the parser’s inner core in the C programming language only after the Perl parser had been written, debugged and found stable, just to improve its performance. The new C module was designed as a drop-in replacement of the Perl parser module in case the performance became an issue.

The parser of the CIF language was implemented with the help of parser generators using bottom-up syntactic analysis algorithms. For Perl, the *Yapp* tool (Desarmenien, 1998 ▸) was used to generate the parser. For C, the parser was implemented using the *Bison* parser generator (Donnely & Stallman, 2015 ▸). These two tools where chosen because they accept nearly identical input file syntax based on the well known *Yacc* parser generator (Johnson, 1975 ▸; Levine, 2009 ▸). Parser generators offer several advantages over writing parsers ‘by hand’ (*e.g.* using a recursive descent method). Since all grammar rules for a parser generator are written concisely and explicitly in a single source file, it is easier to update or extend the language. This is especially useful when CIF format is further developed by the IUCr or when special error correction extensions are introduced. An additional benefit is the readability of the grammar files; since *Yacc* grammars consist of somewhat simplified Backus–Naur Form (BNF) syntax rules, it is easier to check the correspondence of the implemented parser with the CIF grammar, which is published in a BNF-like notation (Hall *et al.*, 2006 ▸; COMCIFS, 2003 ▸).

The parser in C was implemented by porting the *Yapp* grammar to a *Bison* input file and replacing Perl statements by the corresponding C code. The generated C parser can be then used to create standalone programs in C. Alternatively, the parser in C can be bound with many other languages using, for example, the automatic binding generator *SWIG* (Beazley *et al.*, 2015 ▸). We have produced bindings of our *Bison* parser for Perl and Python. Both C and Perl language parsers adhere to the same CIF syntax and produce identical data structures, although they slightly differ in error reporting owing to the employed parser generators. However, a strict syntax for formatting messages and identical internal CIF representations make it possible to substitute the C parser for the Perl parser seamlessly in programs without disrupting other systems that use them. The architecture of *COD::CIF::Parser* in both languages should make it relatively easy to port it to more than 100 computing platforms where C and Perl compilers are available (Hietaniemi, 2010 ▸).

### 2.2. Data structures   

**Figure 1:**
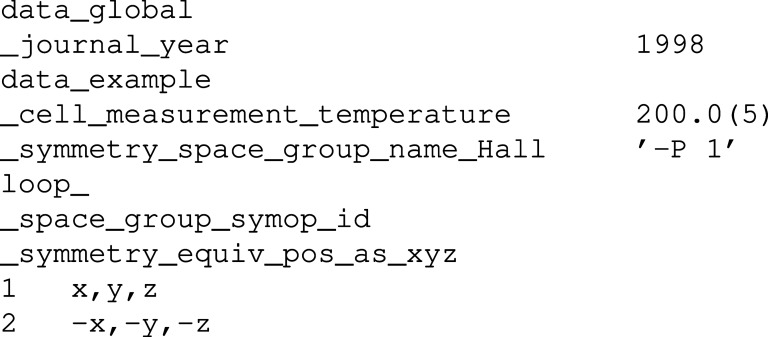
An example of a CIF input for parsing.

**Figure 2:**
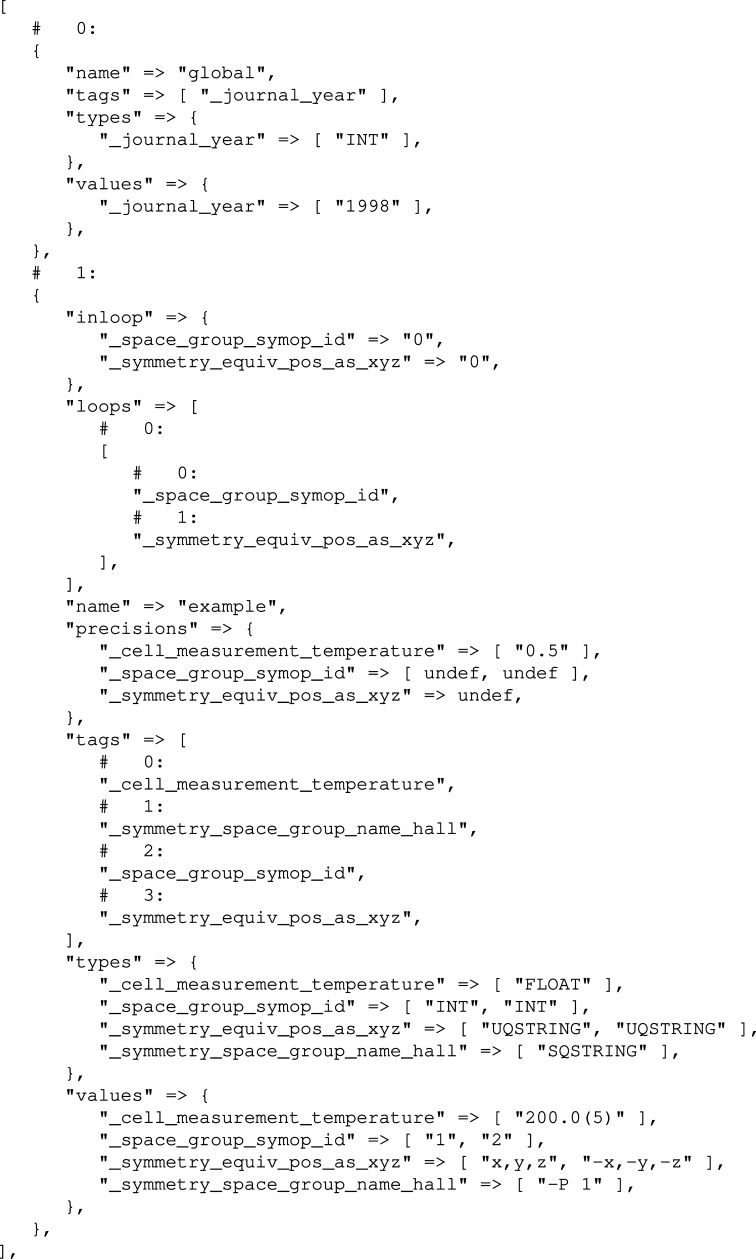
An internal CIF data structure created by *COD::CIF::Parser* after processing the CIF from Fig. 1 ▸.

Each CIF is represented by an array of hash tables (Perl hashes). Each of these hashes represents a single CIF data block. The key–value pairs present in this hash are described in Table 1 ▸. For example, when the CIF from Fig. 1 ▸ is parsed, the parser returns the structure depicted in Fig. 2 ▸. A more general description of a hash constructed from a CIF data block can be found in Appendix *A* [App appa] and in the supplementary file po5052sup1.txt.

Numeric values are stored as strings with possible standard uncertainties, leaving the conversion to an application. Thus, no numeric precision is lost during the parsing. Comments in the CIF are ignored, as they should not contain any important machine-readable information. The same convention is followed by *PyCIFRW*. The absence of language constructs for comments in some widely used data formats, such as JSON, additionally justifies this decision.

### 2.3. Error detection and correction   

*COD::CIF::Parser* is designed to detect and report syntax errors as well as to apply heuristics to fix the most common ones: add missing data_ headers, resolve duplicate tags, insert missing quotes *etc*. The error detection is implemented *via* extended CIF grammar rules that recognize erroneous constructions. For example, all CIF values occurring before the header of the first data block are recognized as CIF data values and can be ignored as a possibly malformed comment when parsed in the error correcting mode. The full list of recognizable errors and their treatments is provided below; each of the heuristics can be turned on and off using corresponding parser options (given in bold):

(*a*) Stray CIF values before the first data block – ignored (**fix_data_header**).

(*b*) No data_ header – ignored (**fix_data_header**).

(*c*) Stray CIF values after the data block name – appended to the data block name (**fix_datablock_names**).

(*d*) Duplicate data items – if all data items report the same value, only one data item is retained (**fix_duplicate_tags_with_same_values**). The duplicate data name error is not corrected if two data items with the same name contain different values, to prevent incorrect interpretation of the input.

(*e*) Items with duplicate data names, where only one data item contains a known value (*i.e.* a value that is not equal to a single question mark or a single period) – only the data item with the known value is retained (**fix_duplicate_tags_with_empty_values**).

(*f*) More than one value for a single non-loop tag – all values taken as quoted (**fix_string_quotes**).

(*g*) Unquoted strings starting with an opening square bracket (‘[’) – treated as single-quoted strings (**allow_uqstring_brackets**).

(*h*) ^Z symbols – removed (**fix_ctrl_z**).

(*i*) Other non-ASCII symbols – these are encoded as XHTML character references (Pemberton *et al.*, 2000 ▸) (**fix_non_ascii_symbols**).

(*j*) Missing single or double closing quote – an appropriate quote is inserted (**fix_missing_closing_single_quote** and **fix_missing_closing_double_quote**).

Correction of all of the aforementioned errors can be enabled with the **fix_all** option. The errors and the applied changes are reported to the standard error channel, using both human- and machine-readable format, as described in §2.4. Also, the total number of errors can be requested from the parser.

### 2.4. Error reporting   

When an issue is detected during the processing of a file, most programs issue a message to inform the user about the nature of the problem and how to fix it if necessary. Usually, in a command line environment under Unix or Windows operating systems, a command line program writes out error messages in an informal style, intended mostly for human readers. Our *cod-tools* package, however, is also meant to be integrated into larger systems, such as the COD data deposition web server. In such systems, other programs must analyse the output of the CIF parser, including error messages, and take appropriate actions. We have therefore found that a strict formal specification of the error message format is helpful. Since program composition is common in Unix-type systems, our solution might have broader applicability outside *cod-tools* and the CIF environment.

To aid understanding of the error message format, we have composed a formal grammar describing this format and encoded it in the Extended BNF (EBNF) form (ISO, 1996 ▸). This grammar is provided in Appendix *B* [App appb] in a formatted form and in the supplementary file po5052sup2.txt as a computer-readable ASCII file. It can be readily used by software authors to further develop error message formatters and analysers. We need, however, to make sure that the presented grammar is complete, is unambiguous and indeed describes the intended language. To perform computer-aided verification of these properties, we have employed an EBNF parser and analyser. Since we could not find a free EBNF parser on-line, we have implemented a simple BNF and EBNF parser using the *Grammatica* (Cederberg, 2015 ▸) parser generator. *Grammatica* was chosen because it is one of the few parser generators that allows the grammar and the processing code to be kept in separate files. It generates output files in Java, and therefore the EBNF processors were written in that language. (The BNF and EBNF parsers are available as a *grammatiker* package at svn://saulius-grazulis.lt/grammatiker.)

We transform the grammar of error messages (provided in the supplementary file po5052sup2.txt) into a *Grammatica* input file, from which a Java parser can be generated. This transformation makes sure that all grammar rules are properly defined; further processing with *Grammatica* makes sure that the grammar can be interpreted unambiguously and is suitable for an automated parser generator. The generated parser can then be used to check that the error messages from our software tools conform to this definition and are parsed correctly.

**Figure 3:**
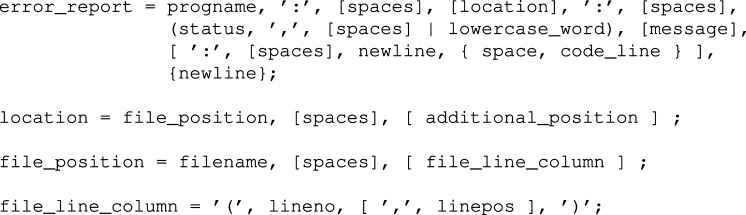
The top-level grammar rules defining error message syntax for *COD::CIF::Parser*.

The meaning of the syntactic components in error messages should be clear from the grammar rule names. Fig. 3 ▸ lists the top-level rules of the error message syntax. Here, the progname is the name of the program that generated the message. The filename is the name of the file in which the error was detected. The file name may be augmented with the optional line and column numbers (lineno and linepos) in parentheses. Additional information about the error position may follow; *cod-tools* package programs output the data block name when processing CIFs. The message text (message) contains a human-readable description of the problem. If applicable, the message text is preceded by the problem severity level (status):

(*a*) ERROR indicates an unrecoverable situation, rendering the output of the program unusable.

(*b*) WARNING indicates that the output of the program can be processed further but may contain results that were not intended in the current situation and therefore should be treated with additional care.

(*c*) NOTE provides an informative message indicating the correct output data; such a message may be dismissed and the processing should proceed.

**Figure 4:**
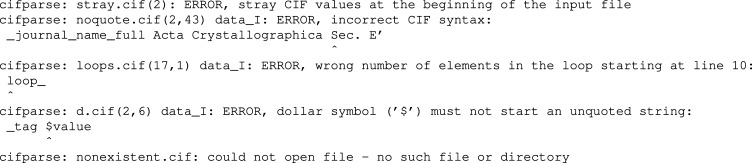
Examples of *COD::CIF::Parser* diagnostic messages.

Where appropriate, messages about syntax errors are followed by an excerpt from the original file (code_line). The error causing token is marked by a caret (‘^’) symbol. Examples of error messages generated according to this grammar are presented in Fig. 4 ▸.

Since certain symbols are used as delimiters of message parts, they are not permitted in the message text or file names. As follows from the EBNF grammar, file names, for example, may not contain colons (‘:’) and parentheses, and message text may not contain colons. Since, in general, these characters may be present in file names or in the text, we need to escape these characters by some special sequences. Although the provided grammar does not specify any particular escaping method, in the *cod-tools* package we use the XHTML character entity references as a compromise between the simplicity of escaping/unescaping algorithms and human readability. Thus, any text can be encoded in an error message without a loss of information, with acceptable appearance for human readers, and with the possibility to parse the error messages unambiguously by computer algorithms.

### 2.5. Writing CIFs   

Since the data structure of a parsed CIF is enough to reconstruct the initial data, development of CIF writing procedures is straightforward. The appropriate type for a data value can always be found: values containing spaces are enclosed by quotes, and values that can not be expressed as quoted strings are stored in text fields. Although there are certain values that can not be placed even in text fields, in §2.6 we present several ways to convert CIFs into universal data carriers. We have implemented CIF writing in a Perl module, *COD::CIF::Tags::Print*, of the *cod-tools* package, which was able to perform successful round-trips for its own written CIFs: a program was able to write out a CIF that can be read again by the *COD::CIF::Parser* and to produce exactly the same data structure that had been used for writing.

### 2.6. Folding and prefixing   

CIF 1.1 format files have a potential to be used as ‘data containers’ for any data presented as key–value pairs, in a manner similar to that of JSON. Such a feature would be useful to embed input and output files of data processing software in CIF, as is done by *SHELXL2014* (Sheldrick, 2015 ▸). However, restrictions on the character set and the line length together with the non-nestable nature of CIF text fields (lines inside text field must not start with a semicolon, as such a construction is used to mark the termination of a text field) limit this possibility. We have implemented means to bypass these limitations in *COD::CIF::Parser* and *COD::CIF::Tags::Print*. The non-ASCII characters are converted to XHTML character references, as described in §2.3. The long line folding/unfolding protocol is implemented as described in the IUCr’s CIF working specification (Hall *et al.*, 2006 ▸). In addition to this, nesting of CIFs in the text fields was made possible by prefixing/unprefixing the con­tents of the text fields. This feature was proposed to the Committee for the Maintenance of the CIF Standard (COM­CIFS) and is planned to be implemented in the upcoming CIF 2.0 format (Bollinger, 2011 ▸; Bernstein *et al.*, 2016 ▸). However, the prefixing algorithm, when applied to arbitrary text values, produces valid CIF 1.1 format files. Therefore we can treat prefixed data fields in CIF 1.1 as an application convention, even if it is not mandated by the CIF 1.1 standard. This extension permits us to store arbitrary data values in CIF text fields in a way that is compatible with the CIF 1.1 standard and is recognizable by the prefix-aware software. If unprefixing or unfolding are not desired, either or both functions can be turned off using parser options **do_not_unprefix_text** and **do_not_unfold_text** in the CIF parser.

## 3. Results   

The most prominent application of our CIF parser is the maintenance and enhancement of the COD (Gražulis *et al.*, 2009 ▸). The use of error correction functionality allows several objectives to be achieved.

On one hand, the strict mode of *COD::CIF::Parser* ensures that all CIFs in the COD adhere to the CIF description provided by the IUCr. For this purpose, the *cifparse* program was written entirely in C to provide the command line interface for the parser. It was then used to check every CIF stored in the COD Subversion repository for syntactic correctness. The *cifparse* program is now run in the Subversion repository from a pre-commit hook. This way, we can be sure that files with wrong syntax will not find their way into the COD. The same check is performed by the COD data deposition web site, which checks syntactic correctness prior to deposition. Only when the CIF syntax is correct and the file is machine readable do we perform further semantic checks.

On the other hand, when accepting files for deposition from researchers or from published sources, such strict adherence to the CIF format may do more harm than good. There are a number of deviations from the CIF standard, such as missing data headers, forgotten closing quotes or data block names with embedded spaces. All these discrepancies can be corrected automatically, and we argue that all human readers will agree on what the intended message of the data file was and what the corrections should be, if that published file can be understood at all. Hence the error correction mode of our *COD::CIF::Parser* turns out to be very useful, doing a great deal of tedious work automatically without bothering human users. Of course, in an interactive session all such corrections are indicated in NOTEs (see §2.4) and presented for a COD depositor to review, and in both automatic and interactive data collectors these issues are registered in log files for further inspection. Such assessment of the NOTEs, presented in the web deposition site or pooled in the log files, requires much less work than manual typing of the corrections, thus significantly reducing COD workload and cost. The employment of our parser in the COD allowed us to increase the number of entries there beyond 300 000 in a highly automated way and supports the ongoing data curation activities in the COD database. Other uses of the *COD::CIF::Parser* module are the following:

(*a*) **Format conversion** – *COD::CIF::Parser* enables the conversion of CIF format to other widely used lossless data formats (*i.e.* JSON, as implemented in *cif2json* from *cod-tools*) or field-specific formats (*i.e.* input formats of DFT codes).

(*b*) **Crystallographic computations** – *COD::CIF::Parser* allows one to read CIF data into C, Perl, Python and potentially Fortran programs. We have harnessed our parser in programs that compute molecular structures from CIFs and analyse molecule symmetry (Gražulis *et al.*, 2015 ▸).

(*c*) **Validation** – the IUCr describes the protocol to validate CIFs against dictionaries, and dictionaries against their Dictionary Definition Language (DDL) dictionaries.

**Figure 5:**
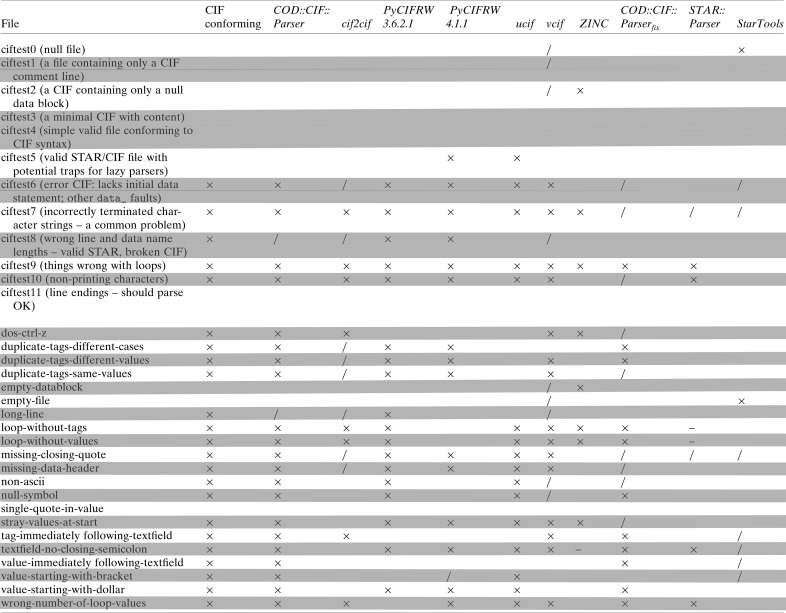
Tests of CIF and STAR parsers. Crosses (‘
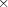
’) denote detected syntax errors, slashes (‘
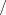
’) denote warnings, and dashes (‘–’) mark special cases when programs hang for an indefinite amount of time and have to be stopped manually. In the column ‘CIF conforming’ crosses mark files that do not conform to CIF syntax.

For the comparison of parsing behaviour of various CIF parsers, we have performed syntactical analysis of two sets of synthetic CIFs. The first set is the ‘trip’ test suite for *vcif* and the second is a subset of our own test cases. We have chosen to test a subset of the most popular (according to Google Search) open-source command-line-compatible CIF parsers, *cif2cif* (version 2.0.0), *PyCIFRW* (versions 3.6.2.1 and 4.1.1), *ucif* (revision 23314), *vcif* (version 1.2), *ZINC* (version 1.12) and our CIF parsers (version 1.0). We have also investigated the *vcif2* (version 0.9.3.1) CIF parser, but have decided to exclude it from analysis, as it seems that the parser’s parsing time grows quadratically with the size of the CIF text fields. Since CIF is a subset of the STAR format and STAR parsers are able to parse CIF, two STAR parsers, *STAR::Parser* (version 0.59) and *StarTools* (version 0.2.0), were also included in the list of tested parsers. As our intent was to check the default behaviour of parsers, no command line options or arguments were used, except for *COD::CIF::Parser*.^2^ The results of the analysis are summarized in Fig. 5 ▸.

To compare the performance of parsers, 350 598 CIFs were parsed from the COD (all entries from revision 170418, totalling ∼16 G) on an unloaded computer with 31 GB of RAM and 16 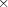 Intel(R) Xeon(R) CPU E5-2450 v2 @ 2.50 GHz, under Debian GNU/Linux 8.2 (jessie), using gcc version 4.9.2, Perl version 5.20.2 and Python version 2.7.9. Wall clock timings are presented in Table 2 ▸. Our tests indicate that our C parser is among the fastest in the field, while at the same time it recognizes most of the CIF features as mandated by the IUCr grammar.

So far, *COD::CIF::Parser* only accepts CIF 1.1 format input. Since the parser is generated from a grammar closely resembling the BNF, the parser can be extended by amending its source code in case COMCIFS implements extensions of the CIF format. Notably, the development of the CIF 2.0 format is under way (Bernstein *et al.*, 2016 ▸). When the details of the CIF 2.0 standard are set, the parser can be extended, when necessary, to accommodate this format as well. Ideally, this should be done by adding further productions to the CIF grammar. This would maintain backwards compatibility with the previous CIF 1.*x* format and make sure that a single parser can read both old format files from the archives and the newly arriving extended syntax files – a feature very important for the long-term archiving format. It turns out, however, that the CIF 2.0 extensions are backwards incompatible with the CIF 1.*x* series of formats, and therefore a second grammar and a second parser will have to be maintained. This is possible to achieve by transforming the defining grammar of our parsers, but the construction of the second set of parsers might add more cost and require more effort than writing the original parser, since the software development costs grow worse than linearly with the system size (Boehm, 1981 ▸).

## 4. Discussion   

To facilitate data exchange between different computer programs and between different laboratories, an agreement on a common data format or formats and strict adherence to the definition of these formats is needed. Deviations cause unnecessary delays in data processing, a need for extra human intervention and, in the worst case, corrupt data and incorrect results. We therefore strive to implement a CIF parser to the letter of the IUCr CIF 1.1 documentation. Fig. 5 ▸ shows that our parser in strict mode accepts all conforming files from the *vcif* ‘trip’ suite and reports all non-conforming files. We also carry out extra tests to check additional features of the CIF grammar.

There is one area, however, where we decided to be slightly less strict than the IUCr specification. In our parser, we tolerate arbitrarily long lines, and report them only if this check is explicitly requested. This is justified by the fact that the programming languages we use (Perl and C) can seamlessly process strings of arbitrary length, and limiting line length is in this case an extra burden. Reading long lines does not lose or corrupt data in any way; conversely, limiting line length and discarding trailing symbols might lead to data loss. To our knowledge, all other modern programming systems (*e.g.* Perl, Python, Java and Julia to name just a few) support dynamic strings, and therefore programs written in these languages should behave in a similar way. There is therefore a drawback to rejecting an otherwise correct file just because it has long lines, and the detection of these lines gives no benefit unless we check our files for interaction with old style Fortran programs. We thus decided to make our CIF reader as permissive as possible, while of course writing out files as strictly adhering as possible to the CIF 1.1 specification. Such a policy guarantees that our tools are compatible with their own output, their output is suitable for the largest number of programs, and the tools themselves are capable of reading the maximum number of inputs.

It is interesting to compare how different parsers behave on various test sets of CIFs. Most CIF parsers seem to be able to parse the correct CIFs and report the erroneous ones (Fig. 5 ▸) from the *vcif* test suite. In some special cases, however, certain parsers exhibit different behaviour; for instance, *ZINC* fails at test case 2 (described as containing a null datablock), and both *PyCIFRW* (version 4.1.1 only) and *ucif* fail at test case 5 (described as containing potential traps for lazy parsers). It should be noted, however, that *PyCIFRW* 3.6.2.1 is able to parse test case 5. The parsers *cif2cif*, *COD::CIF::Parser*, *PyCIFRW* and *vcif* report data item names that exceed 80 characters at test case 8, with *PyCIFRW* even halting after discovering an exceedingly long data item name.

Our own test cases seem to elicit even more diverse reactions among CIF parsers. The NULL symbol, which is not allowed in the CIF syntax, is reported as an error by our parsers, *PyCIFRW* 3.6.2.1 and *ucif*; *vcif* reports the symbol with a warning. The control symbol ^Z seems to be undetected by *PyCIFRW* and *ucif*, even though it is not allowed by the CIF syntax. The parsers *cif2cif*, *PyCIFRW* 3.6.2.1, *vcif* and *ZINC* do not report an unquoted CIF value starting with an opening bracket (‘[’). In addition, *cif2cif*, *vcif* and *ZINC* accept values starting with a dollar sign (‘$’). These two symbols are reserved and thus they are not allowed to start CIF data items. All CIF parsers except *PyCIFRW* 4.1.1 are able to detect and report CIF loop constructions without either tags or values, whereas STAR parsers behave differently: *STAR::Parser* appears to get caught in an infinite loop and *StarTools* does not report them at all. CIF tags and values following text fields without intermediate white space are detected as errors by *COD::CIF::Parser* and *StarTools* only; *cif2cif* and *vcif* detect only tags following text fields. *ZINC* proves to be a quite robust, low-level tool, being able to continue parsing even after running into non-ASCII symbols, missing closing quotes and files with missing data headers. Syntactic and semantic error detection is then left for higher-level tools. However, *ZINC* gets caught in an infinite loop after encountering a text field without a closing semicolon and has to be stopped manually. All other parsers are able to detect this kind of error and stop gracefully, with the exception of *cif2cif*, which adds a terminating semicolon without notice. A wrong number of CIF loop elements – more a semantic than a syntactic error – is reported by all CIF parsers but *PyCIFRW* 3.6.2.1 and *ZINC*. Interestingly enough, these parsers employ heuristics to remove overflowing tags or values. For the purposes of the COD, however, such behaviour is too dangerous since it can lead to the loss of data. Therefore *COD::CIF::Parser* regards incomplete loop_ tables as fatal errors, like *vcif* does.

Such diversity of parser reactions towards input files probably reflects different needs and tasks, on one hand, and different engineering trade-offs chosen by various authors, on the other. For example, in the COD where the purpose is to collect as much reliable data as possible from supplementary data of publications and from user-provided files, the CIF reader must be permissive; since extra-long lines do not corrupt information in CIFs, we adhere to the limitations of data item and line lengths when generating output, but not when processing the input. Other situations, however, might require different emphasis.

Finally, *COD::CIF::Parser* with extended CIF syntax (entitled *COD::CIF::Parser* 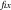 in Fig. 5 ▸) allows us to repair most of the CIFs from the *vcif* test suite, as well as some erroneous CIFs from our own test suite. Such behaviour proved very useful in dealing with near-CIF input; our experience shows that files of this kind are encountered even in the data from peer-reviewed publications. Overall, the diverse behaviour of existing CIF parsers and various needs of different applications demonstrate the utility of having several parsers available – on one hand, comparison of different parser behaviour allows us to spot bugs in our own design; on the other hand, a parser with the most suitable trade-offs (speed, manageability, dependencies, compatibility with programming languages in use) can be chosen when needed. Hitting the sweet spot of engineering trade-offs suitable for the COD was one of the motivations to build a CIF parser for Perl.

## 5. Conclusion   

A CIF format parser was implemented in the Perl programming language and optimized by providing a C library with Perl and Python bindings. Our tests show that the resulting code is one of the fastest and the most accurate CIF parsers in the field. Comparing its output with that of other parsers was instrumental in testing and debugging our parsers, as different parsers exhibit different behaviour in some test cases. We must note that such differences do not necessarily indicate errors since different behaviour might be useful in different situations. In particular, our parser’s special error correcting mode is necessary to ingest crystallographic data in near-CIF format from various external sources. Thus, our parser proved useful in managing large worldwide collections of crystallographic data. In addition, fast performance and the possibility to call the parser from different programs in the Perl, C and Python languages allows one to employ it in various crystallographic programs, and we hope that it will enable easier data exchange between researchers. The *COD::CIF::Parser* parser can be downloaded as part of the *cod-tools* software package (http://www.crystallography.net/archives/2015/software/cod-tools/cod-tools-1.0.tbz2) under the GPL2 free software license. The package is also available as part of the supporting information for this article.

## Supplementary Material

Internal CIF data representation. DOI: 10.1107/S1600576715022396/po5052sup1.txt

EBNF grammar. DOI: 10.1107/S1600576715022396/po5052sup2.txt

cod-tools package. DOI: 10.1107/S1600576715022396/po5052sup3.tgz

## Figures and Tables

**Table 1:** Key–value pairs of a hash that represents a single CIF data block as constructed by *COD::CIF::Parser*

Key	Value
name	Scalar. String denoting the name of a CIF data block.
tags	Array. Lower-cased data names present in the CIF data block.
values	Hash table. Keys are equal to the values of the tags array. Values are arrays containing values for each data item.
types	Hash table. Keys are equal to the values of the tags array. Values are arrays containing lexically derived data types for each data item.
precisions	Hash table. Keys are equal to the values of the tags array. Values are arrays containing standard uncertainties for each data item.
loops	Array of arrays. Each inner array corresponds to a loop from the CIF data block and contains a list of tags present in the loop.
inloop	Hash. Keys are equal to the values of the tags array. Values correspond to indices of the outer loops array. It is used as an index to optimize tag-in-loop related searches.

**Table 2:** Total parsing time of 350 598 CIFs from the COD

Parser	Run time (min)
*cif2cif*	19.73
*COD::CIF::Parser*	3.95
*COD::CIF::Parser* 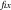	3.62
*PyCIFRW-3.6.2.1*	39.75
*PyCIFRW-4.1.1*	3062.22
*ucif*	5.15
*vcif*	2.68
*ZINC*	3.67

## Footnotes

1 Available under the GPL2 free software license at svn://www.crystallography.net/cod-tools/tags/v1.0, this paper refers to version 1.0 (source revision 4339), which can be also obtained from http://www.crystallography.net/archives/2015/software/cod-tools/cod-tools-1.0.tbz2.

2 For *COD::CIF::Parser* we have explicitly used command line option --report-long-items to turn on line and data item length checks, which are turned off by default.

## Appendix A Internal CIF data representation

The internal representation of a parsed CIF produced by *COD::CIF::Parser*:

## Appendix B EBNF grammar of the messages

The EBNF grammar used to format error and warning messages of *COD::CIF::Parser* and the *cod-tools* package:
