# Supplementary material for: COD::CIF::Parser: an error-correcting CIF parser for the Perl language
Source: J Appl Crystallogr. 2016 Feb 1;49(Pt 1):292–301. doi: 10.1107/S1600576715022396 (PMC4762566; doi:10.1107/S1600576715022396)
Supplement: Supplementary file 3 [file j-49-00292-sup3.tgz › cod-tools-1.0/src/components/codcif/data/IUCr/CIFTEST_2.1/CIFTEST_README.html]

CIFTEST2


## CIFTEST2 based in part on the package trip by B. McMahon

### Release 2.1

© Copyright 2005, 2006 by Georgi Todorov
  
Work supported in part by the U.S. National Science Foundation
under grants DBI-0315281 and DBI-0610407, by the U.S. Department
of Energy under grants ER63601-1021466-0009501 and ER64212-1027708-0011962
and by the International Union of Crystallography.


---

### COPYING

This program is free software; you can redistribute it and/or modify it under the terms of the
GNU General Public License (GPL) as published by the Free Software Foundation; either version 2 of
the License, or (at your option) any later version.

This program is distributed in the hope that it will be useful, but **WITHOUT ANY WARRANTY;
without even the implied warranty of MERCHANTABILITY or FITNESS FOR A PARTICULAR PURPOSE**.
See the GNU General Public License for more details.

You should have received a copy of the GNU General Public License along with this program;
if not, write to the Free Software Foundation, Inc., 59 Temple Place - Suite 330, Boston, MA 02111-1307, USA.

### Installing CIFTEST2

Download the tarball CIFTEST.tar.gz and unpack it
to create the CIFTEST\_2.1 directory in the current directory. See the following
material to then use what you have unpacked.

### What is CIFTEST2

CIFTEST2 is a test kit for CIF software. The current version supports
vcif, CIFFOLD, cif2cif, cif2xml, ciftbx, cif2pdb and cyclops. It is a shell script
(sh) assuming the following directory tree structure:

```
|-- CIFTEST_2.1.tar.gz -> ../CIFTEST_2.1.tar.gz
|-- CIFTEST_README.html
|-- COPYING
|-- README.odt
|-- README.txt
|-- args
|   |-- cif2cbf
|   |   |-- c2ctest0.cif.args
|   |   |-- c2ctest1.cif.args
|   |   |-- ciftest0.cif.args
|   |   |-- ciftest1.cif.args
|   |   |-- ciftest10.cif.args
|   |   |-- ciftest11.cif.args
|   |   |-- ciftest2.cif.args
|   |   |-- ciftest3.cif.args
|   |   |-- ciftest4.cif.args
|   |   |-- ciftest5.cif.args
|   |   |-- ciftest6.cif.args
|   |   |-- ciftest7.cif.args
|   |   |-- ciftest8.cif.args
|   |   `-- ciftest9.cif.args
|   |-- ciffold
|   |   |-- 1ejg.cif.args
|   |   |-- longcomments.cif.args
|   |   `-- longtext.cif.args
|   |-- ciftbx
|   `-- vcif
|       |-- ciftest0.args
|       |-- ciftest1.args
|       |-- ciftest10.args
|       |-- ciftest11.args
|       |-- ciftest2.args
|       |-- ciftest3.args
|       |-- ciftest4.args
|       |-- ciftest5.args
|       |-- ciftest6.args
|       |-- ciftest7.args
|       |-- ciftest8.args
|       `-- ciftest9.args
|-- ciftest2
|-- descriptions
|   |-- cif2cbf
|   |   |-- c2ctest0.cif.purpose
|   |   |-- c2ctest1.cif.purpose
|   |   |-- ciftest0.cif.purpose
|   |   |-- ciftest1.cif.purpose
|   |   |-- ciftest10.cif.purpose
|   |   |-- ciftest11.cif.purpose
|   |   |-- ciftest2.cif.purpose
|   |   |-- ciftest3.cif.purpose
|   |   |-- ciftest4.cif.purpose
|   |   |-- ciftest5.cif.purpose
|   |   |-- ciftest6.cif.purpose
|   |   |-- ciftest7.cif.purpose
|   |   |-- ciftest8.cif.purpose
|   |   `-- ciftest9.cif.purpose
|   |-- ciffold
|   |-- ciftbx
|   `-- vcif
|       |-- ciftest0.purpose
|       |-- ciftest1.purpose
|       |-- ciftest10.purpose
|       |-- ciftest11.purpose
|       |-- ciftest2.purpose
|       |-- ciftest3.purpose
|       |-- ciftest4.purpose
|       |-- ciftest5.purpose
|       |-- ciftest6.purpose
|       |-- ciftest7.purpose
|       |-- ciftest8.purpose
|       `-- ciftest9.purpose
|-- dictionaries
|   |-- cif_cml.dic
|   |-- cif_core.dic
|   `-- cif_mm.dic
|-- errs
|   |-- cif2cbf
|   |-- ciffold
|   |-- ciftbx
|   `-- vcif
|-- gens
|   |-- cif2cbf
|   |-- ciffold
|   |-- ciftbx
|   `-- vcif
|-- origs
|   |-- cif2cbf
|   |   |-- c2ctest0.cif
|   |   |-- c2ctest1.cif
|   |   |-- cif_cml.dic -> ../../dictionaries/cif_cml.dic
|   |   |-- cif_core.dic -> ../../dictionaries/cif_core.dic
|   |   |-- cif_mm.dic -> ../../dictionaries/cif_mm.dic
|   |   |-- ciftest0.cif
|   |   |-- ciftest1.cif
|   |   |-- ciftest10.cif
|   |   |-- ciftest11.cif
|   |   |-- ciftest2.cif
|   |   |-- ciftest3.cif
|   |   |-- ciftest4.cif
|   |   |-- ciftest5.cif
|   |   |-- ciftest6.cif
|   |   |-- ciftest7.cif
|   |   |-- ciftest8.cif
|   |   `-- ciftest9.cif
|   |-- ciffold
|   |   |-- 1ejg.cif
|   |   |-- 1zrt.cif
|   |   |-- longcomments.cif
|   |   `-- longtext.cif
|   |-- ciftbx
|   |   |-- 1ace.cif
|   |   |-- 1crn.cif
|   |   |-- 1cro.cif
|   |   |-- 1cwp.cif
|   |   |-- 1hyh.cif
|   |   |-- 1zrt.cif
|   |   |-- 2ace.cif
|   |   |-- 4hir.cif
|   |   |-- 4ins.cif
|   |   |-- 5hvp.cif
|   |   |-- ADH041.cif
|   |   |-- BDL001.cif
|   |   |-- BDLB13.cif
|   |   |-- DDF040.cif
|   |   |-- STARDICT
|   |   |-- c2ctest0.cif
|   |   |-- c2ctest1.cif
|   |   |-- cif_cml.dic -> ../../dictionaries/cif_cml.dic
|   |   |-- cif_core.dic -> ../../dictionaries/cif_core.dic
|   |   |-- cif_mm.dic -> ../../dictionaries/cif_mm.dic
|   |   |-- mtest.prt
|   |   |-- qtest.cif
|   |   |-- qtest.req
|   |   |-- test.cif
|   |   |-- test.req
|   |   `-- xtalt2.cif
|   `-- vcif
|       |-- ciftest0
|       |-- ciftest1
|       |-- ciftest10
|       |-- ciftest11
|       |-- ciftest2
|       |-- ciftest3
|       |-- ciftest4
|       |-- ciftest5
|       |-- ciftest6
|       |-- ciftest7
|       |-- ciftest8
|       `-- ciftest9
`-- outs
    |-- cif2cbf
    |   |-- c2ctest0.cif.result
    |   |-- c2ctest1.cif.result
    |   |-- ciftest0.cif.result
    |   |-- ciftest1.cif.result
    |   |-- ciftest10.cif.result
    |   |-- ciftest11.cif.result
    |   |-- ciftest2.cif.result
    |   |-- ciftest3.cif.result
    |   |-- ciftest4.cif.result
    |   |-- ciftest5.cif.result
    |   |-- ciftest6.cif.result
    |   |-- ciftest7.cif.result
    |   |-- ciftest8.cif.result
    |   `-- ciftest9.cif.result
    |-- ciffold
    |   |-- 1ejg_out.cif
    |   |-- 1ejg_out2.cif
    |   |-- 1ejg_rebuilt.cif
    |   |-- 1ejg_rebuilt2.cif
    |   |-- 1zrt_out.cif
    |   |-- 1zrt_out2.cif
    |   |-- 1zrt_rebuilt.cif
    |   |-- 1zrt_rebuilt2.cif
    |   |-- longcomments_out.cif
    |   |-- longcomments_out2.cif
    |   |-- longcomments_out2.cif~
    |   |-- longtext_out.cif
    |   `-- longtext_out2.cif
    |-- ciftbx
    |   |-- 1ace.stwid
    |   |-- 1ace.tpdb
    |   |-- 1ace.twid
    |   |-- 1crn.tpdb
    |   |-- 1crn.twid
    |   |-- 1cro.tpdb
    |   |-- 1cro.twid
    |   |-- 1cwp.tpdb
    |   |-- 1cwp.twid
    |   |-- 1hyh.tpdb
    |   |-- 1hyh.twid
    |   |-- 1zrt.tpdb
    |   |-- 1zrt.twid
    |   |-- 2ace.tpdb
    |   |-- 2ace.twid
    |   |-- 4hir.tpdb
    |   |-- 4hir.twid
    |   |-- 4ins.out
    |   |-- 4ins.out.x
    |   |-- 4ins.prt
    |   |-- 4ins.prt.x
    |   |-- 4ins.tpdb
    |   |-- 4ins.twid
    |   |-- 4insuw.out
    |   |-- 4insuw.prt
    |   |-- 4insw.out
    |   |-- 4insw.prt
    |   |-- 5hvp.tpdb
    |   |-- 5hvp.twid
    |   |-- ADH041.tpdb
    |   |-- ADH041.twid
    |   |-- BDL001.tpdb
    |   |-- BDL001.twid
    |   |-- BDLB13.tpdb
    |   |-- BDLB13.twid
    |   |-- DDF040.tpdb
    |   |-- DDF040.twid
    |   |-- c2ctest0.out
    |   |-- c2ctest1.out
    |   |-- cyclops_test.prt
    |   |-- mtest.cyc
    |   |-- mtest.out
    |   |-- mtest.prt
    |   |-- mtest.xml
    |   |-- qtest.out
    |   |-- qtest.prt
    |   |-- test.out
    |   |-- test.prt
    |   |-- testrle.prt
    |   |-- xtalt2.out
    |   |-- xtalt2.out.x
    |   |-- xte29.out
    |   |-- xte29.out.x
    |   |-- xttne9.out
    |   `-- xttne9.out.x
    `-- vcif
        |-- ciftest0.result
        |-- ciftest1.result
        |-- ciftest10.result
        |-- ciftest11.result
        |-- ciftest2.result
        |-- ciftest3.result
        |-- ciftest4.result
        |-- ciftest5.result
        |-- ciftest6.result
        |-- ciftest7.result
        |-- ciftest8.result
        `-- ciftest9.result
```

### BEFORE USING:

Before using CIFTEST v.2 please update the paths in the file "ciftest2".
This can be done by setting the appropriate environment variables:

| Variable | Meaning |
| --- | --- |
| CIFTEST2DIR | path to the CIFTEST2 directory |
| VCIF | full path of vcif program |
| CIF2CIF | full path of cif2cif program |
| CIF2CBF | full path of cif2cbf program |
| CIF2PDB | full path of cif2pdb program |
| CIF2XML | full path to cif2xml program |
| CIFTBX\_EX | full path to CIFtbx tbx\_ex program |
| CIFTBX\_EXM | full path to CIFtbx tbx\_exm program |
| CIFTBX\_TESTRLE | full path to CIFtbx testrle program |
| PREFIX | prefix for the bin directory containing programs to be tested  e.g. /usr/local  only used for programs not specified above |

If you are using one of the sh-style shells, and you have put the programs to be tested
into /usr/local/bin, and will execute ciftest2 from from the installation directory,
you might do:

```
CIFTEST2DIR=.
PREFIX=/usr/local/bin
export CIFTEST2DIR
export PREFIX
```

or, if you use csh, you might do:

```
setenv CIFTEST2DIR .
setenv PREFIX /usr/local/bin
```

If you are trying out, some new version of, say, ciffold\_0.5.4, and installed that binary
as ~/bin/ciffold\_0.5.4, you might override the use of PREFIX, above, for that particular
program with:

```
CIFFOLD=~/bin/ciffold_0.5.4
EXPORT CIFFOLD
```

or

```
setenv CIFFOLD ~/bin/ciffold_0.5.4
```

### USAGE:

```
ciftest2 vcif    -> performs vcif tests
ciftest2 ciffold -> performs CIFFOLD tests
ciftest2 cif2cbf -> performs cif2cbf tests
ciftest2 cif2cif -> performs cif2cif tests
ciftest2 cif2pdb -> performs cif2pdb tests
ciftest2 cif2xml -> performs cif2xml tests
ciftest2 ciftbx  -> performs ciftbx  tests
ciftest2 cyclops -> performs cyclops tests
ciftest2 ciftbx3 -> performs all ciftbx3 tests
ciftest2 all     -> pefroms all tests
ciftest2 clean   -> removes all output and diff files
```

---


Version 1.9.96, G. Todorov, 27 Nov 05  
Version 1.9.96, G. Todorov, 28 Nov 05  
Version 1.9.97, G. Todorov, 30 Nov 05  
Version 2.0, H. J. Bernstein, 30 Jan 06  
Version 2.1, H. J. Bernstein, 3 Sep 06
