# Supplementary material for: COD::CIF::Parser: an error-correcting CIF parser for the Perl language
Source: J Appl Crystallogr. 2016 Feb 1;49(Pt 1):292–301. doi: 10.1107/S1600576715022396 (PMC4762566; doi:10.1107/S1600576715022396)
Supplement: Supplementary file 3 [file j-49-00292-sup3.tgz › cod-tools-1.0/data/AtomProperties/sources/wikipedia/oxidation_states.html]

List of oxidation states of the elements - Wikipedia, the free encyclopedia


# List of oxidation states of the elements

From Wikipedia, the free encyclopedia

Jump to: navigation, search

Main article: Oxidation state

This is a list of known **oxidation states of the chemical elements**, excluding nonintegral values. The most common states appear in bold. The table is based on that of Greenwood and Earnshaw,^[1]^
with additions noted. Oxidation state 0, which occurs for all elements,
is implied by the column with the symbol of the element. The format of
the table, which was devised by Mendeleev in 1889, shows the periodicity of the oxidation states of the elements.^[1]^

## List[edit]

Element

Noble gas

| Element | Negative  oxidation  states | | | | Zero | Positive  oxidation  states | | | | | | | | Notes |
| --- | --- | --- | --- | --- | --- | --- | --- | --- | --- | --- | --- | --- | --- | --- |
|  | *−4* | *−3* | *−2* | *−1* | *0* | *+1* | *+2* | *+3* | *+4* | *+5* | *+6* | *+7* | *+8* |  |
| hydrogen |  |  |  | **−1** | **H** | **+1** |  |  |  |  |  |  |  |  |
| helium |  |  |  |  | **He** |  |  |  |  |  |  |  |  |  |
| lithium |  |  |  |  | **Li** | **+1** |  |  |  |  |  |  |  |  |
| beryllium |  |  |  |  | **Be** | +1 | **+2** |  |  |  |  |  |  | ^[2]^ |
| boron |  |  |  |  | **B** | +1 | +2 | **+3** |  |  |  |  |  |  |
| carbon | **−4** | **−3** | **−2** | **−1** | **C** | **+1** | **+2** | **+3** | **+4** |  |  |  |  |  |
| nitrogen |  | **−3** | −2 | −1 | **N** | +1 | +2 | **+3** | +4 | **+5** |  |  |  |  |
| oxygen |  |  | **−2** | −1 | **O** | +1 | +2 |  |  |  |  |  |  |  |
| fluorine |  |  |  | **−1** | **F** |  |  |  |  |  |  |  |  |  |
| neon |  |  |  |  | **Ne** |  |  |  |  |  |  |  |  |  |
| sodium |  |  |  | −1 | **Na** | **+1** |  |  |  |  |  |  |  |  |
| magnesium |  |  |  |  | **Mg** | +1 | **+2** |  |  |  |  |  |  | ^[3]^ |
| aluminium |  |  |  |  | **Al** | +1 | +2 | **+3** |  |  |  |  |  | ^[4]^ |
| silicon | **−4** | −3 | −2 | −1 | **Si** | +1 | +2 | +3 | **+4** |  |  |  |  |  |
| phosphorus |  | **−3** | −2 | −1 | **P** | +1 | +2 | **+3** | +4 | **+5** |  |  |  |  |
| sulfur |  |  | **−2** | −1 | **S** | +1 | **+2** | +3 | **+4** | +5 | **+6** |  |  |  |
| chlorine |  |  |  | **−1** | **Cl** | **+1** | +2 | **+3** | +4 | **+5** | +6 | **+7** |  |  |
| argon |  |  |  |  | **Ar** |  |  |  |  |  |  |  |  |  |
| potassium |  |  |  | −1 | **K** | **+1** |  |  |  |  |  |  |  |  |
| calcium |  |  |  |  | **Ca** | +1 | **+2** |  |  |  |  |  |  | ^[5]^ |
| scandium |  |  |  |  | **Sc** | +1 | +2 | **+3** |  |  |  |  |  |  |
| titanium |  |  |  | −1 | **Ti** | +1 | +2 | +3 | **+4** |  |  |  |  | ^[6]^ |
| vanadium |  |  |  | −1 | **V** | +1 | +2 | +3 | +4 | **+5** |  |  |  |  |
| chromium |  |  | −2 | −1 | **Cr** | +1 | +2 | **+3** | +4 | +5 | **+6** |  |  |  |
| manganese |  | −3 | −2 | −1 | **Mn** | +1 | **+2** | +3 | **+4** | +5 | +6 | **+7** |  |  |
| iron |  |  | −2 | −1 | **Fe** | +1 | **+2** | **+3** | +4 | +5 | **+6** |  |  | ^[7]^ |
| cobalt |  |  |  | −1 | **Co** | +1 | **+2** | **+3** | +4 | +5 |  |  |  |  |
| nickel |  |  |  | −1 | **Ni** | +1 | **+2** | +3 | +4 |  |  |  |  |  |
| copper |  |  |  |  | **Cu** | +1 | **+2** | +3 | +4 |  |  |  |  |  |
| zinc |  |  |  |  | **Zn** | +1 | **+2** |  |  |  |  |  |  | ^[8]^ |
| gallium |  |  |  |  | **Ga** | +1 | +2 | **+3** |  |  |  |  |  |  |
| germanium | **−4** | −3 | −2 | −1 | **Ge** | +1 | **+2** | +3 | **+4** |  |  |  |  | ^[9]^ |
| arsenic |  | **−3** |  |  | **As** | +1 | +2 | **+3** |  | **+5** |  |  |  | ^[10]^ |
| selenium |  |  | **−2** |  | **Se** | +1 | **+2** |  | **+4** |  | **+6** |  |  | ^[11]^ |
| bromine |  |  |  | **−1** | **Br** | **+1** | **+2** | **+3** | +4 | **+5** |  | **+7** |  |  |
| krypton |  |  |  |  | **Kr** |  | **+2** |  |  |  |  |  |  |  |
| rubidium |  |  |  | −1 | **Rb** | **+1** |  |  |  |  |  |  |  |  |
| strontium |  |  |  |  | **Sr** | +1 | **+2** |  |  |  |  |  |  | ^[12]^ |
| yttrium |  |  |  |  | **Y** | +1 | +2 | **+3** |  |  |  |  |  | ^[13]^^[14]^ |
| zirconium |  |  |  |  | **Zr** | +1 | +2 | +3 | **+4** |  |  |  |  |  |
| niobium |  |  |  | −1 | **Nb** | +1 | +2 | +3 | +4 | **+5** |  |  |  | ^[15]^ |
| molybdenum |  |  | −2 | −1 | **Mo** | +1 | +2 | +3 | **+4** | +5 | **+6** |  |  |  |
| technetium |  | −3 |  | −1 | **Tc** | +1 | +2 | +3 | **+4** | +5 | +6 | **+7** |  |  |
| ruthenium |  |  | −2 |  | **Ru** | +1 | +2 | **+3** | **+4** | +5 | +6 | +7 | +8 |  |
| rhodium |  |  |  | −1 | **Rh** | +1 | +2 | **+3** | +4 | +5 | +6 |  |  |  |
| palladium |  |  |  |  | **Pd** | +1 | **+2** |  | **+4** |  | +6 |  |  | ^[16]^^[17]^ |
| silver |  |  |  |  | **Ag** | **+1** | +2 | +3 | +4 |  |  |  |  | ^[18]^ |
| cadmium |  |  |  |  | **Cd** | +1 | **+2** |  |  |  |  |  |  | ^[19]^ |
| indium |  |  |  |  | **In** | +1 | +2 | **+3** |  |  |  |  |  |  |
| tin | **−4** |  |  |  | **Sn** |  | **+2** |  | **+4** |  |  |  |  |  |
| antimony |  | **−3** |  |  | **Sb** |  |  | **+3** |  | **+5** |  |  |  |  |
| tellurium |  |  | **−2** |  | **Te** |  | **+2** |  | **+4** | +5 | **+6** |  |  |  |
| iodine |  |  |  | **−1** | **I** | **+1** |  | **+3** | +4 | **+5** |  | **+7** |  | ^[20]^ |
| xenon |  |  |  |  | **Xe** | +1 | **+2** |  | **+4** |  | **+6** |  | +8 | ^[21]^ |
| caesium |  |  |  | −1 | **Cs** | **+1** |  |  |  |  |  |  |  |  |
| barium |  |  |  |  | **Ba** |  | **+2** |  |  |  |  |  |  |  |
| lanthanum |  |  |  |  | **La** |  | +2 | **+3** |  |  |  |  |  |  |
| cerium |  |  |  |  | **Ce** |  | +2 | **+3** | **+4** |  |  |  |  |  |
| praseodymium |  |  |  |  | **Pr** |  | +2 | **+3** | +4 |  |  |  |  |  |
| neodymium |  |  |  |  | **Nd** |  | +2 | **+3** | +4 |  |  |  |  | ^[22]^ |
| promethium |  |  |  |  | **Pm** |  | +2 | **+3** |  |  |  |  |  | ^[23]^ |
| samarium |  |  |  |  | **Sm** |  | +2 | **+3** |  |  |  |  |  |  |
| europium |  |  |  |  | **Eu** |  | **+2** | **+3** |  |  |  |  |  |  |
| gadolinium |  |  |  |  | **Gd** | +1 | +2 | **+3** |  |  |  |  |  |  |
| terbium |  |  |  |  | **Tb** | +1 | +2 | **+3** | +4 |  |  |  |  | ^[24]^ |
| dysprosium |  |  |  |  | **Dy** |  | +2 | **+3** | +4 |  |  |  |  | ^[25]^ |
| holmium |  |  |  |  | **Ho** |  | +2 | **+3** |  |  |  |  |  | ^[26]^ |
| erbium |  |  |  |  | **Er** |  | +2 | **+3** |  |  |  |  |  | ^[27]^ |
| thulium |  |  |  |  | **Tm** |  | +2 | **+3** |  |  |  |  |  |  |
| ytterbium |  |  |  |  | **Yb** |  | +2 | **+3** |  |  |  |  |  |  |
| lutetium |  |  |  |  | **Lu** |  |  | **+3** |  |  |  |  |  |  |
| hafnium |  |  |  |  | **Hf** |  | +2 | +3 | **+4** |  |  |  |  |  |
| tantalum |  |  |  | −1 | **Ta** |  | +2 | +3 | +4 | **+5** |  |  |  |  |
| tungsten |  |  | −2 | −1 | **W** | +1 | +2 | +3 | **+4** | +5 | **+6** |  |  |  |
| rhenium |  | −3 |  | −1 | **Re** | +1 | +2 | +3 | **+4** | +5 | +6 | +7 |  |  |
| osmium |  |  | −2 | −1 | **Os** | +1 | +2 | +3 | **+4** | +5 | +6 | +7 | +8 | ^[28]^ |
| iridium |  | −3 |  | −1 | **Ir** | +1 | +2 | **+3** | **+4** | +5 | +6 | +7 | +8 | ^[29]^^[30]^^[31]^ |
| platinum |  |  | −2 | −1 | **Pt** | +1 | **+2** | **+3** | **+4** | +5 | +6 |  |  | ^[32]^^[33]^ |
| gold |  |  |  | −1 | **Au** | +1 | +2 | **+3** |  | +5 |  |  |  |  |
| mercury |  |  |  |  | **Hg** | **+1** | **+2** |  | +4 |  |  |  |  | ^[34]^ |
| thallium |  |  |  | −1 | **Tl** | **+1** |  | **+3** |  |  |  |  |  | ^[35]^ |
| lead | −4 |  |  |  | **Pb** |  | **+2** |  | **+4** |  |  |  |  |  |
| bismuth |  | −3 |  |  | **Bi** | +1 |  | **+3** |  | +5 |  |  |  | ^[36]^ |
| polonium |  |  | **−2** |  | **Po** |  | **+2** |  | **+4** | +5 | +6 |  |  | ^[37]^ |
| astatine |  |  |  | **−1** | **At** | **+1** |  | +3 |  | +5 |  | +7 |  |  |
| radon |  |  |  |  | **Rn** |  | **+2** |  |  |  | +6 |  |  | ^[38]^^[39]^^[40]^ |
| francium |  |  |  |  | **Fr** | **+1** |  |  |  |  |  |  |  |  |
| radium |  |  |  |  | **Ra** |  | **+2** |  |  |  |  |  |  |  |
| actinium |  |  |  |  | **Ac** |  | +2 | **+3** |  |  |  |  |  | ^[41]^ |
| thorium |  |  |  |  | **Th** |  | +2 | +3 | **+4** |  |  |  |  |  |
| protactinium |  |  |  |  | **Pa** |  | +2 | +3 | +4 | **+5** |  |  |  | ^[42]^ |
| uranium |  |  |  |  | **U** |  | +2 | +3 | +4 | +5 | **+6** |  |  | ^[43]^ |
| neptunium |  |  |  |  | **Np** |  |  | +3 | +4 | **+5** | +6 | +7 |  |  |
| plutonium |  |  |  |  | **Pu** |  |  | +3 | **+4** | +5 | +6 | +7 | +8 | ^[44]^ |
| americium |  |  |  |  | **Am** |  | +2 | **+3** | +4 | +5 | +6 | +7 |  | ^[45]^ |
| curium |  |  |  |  | **Cm** |  | +2 | **+3** | +4 |  | +6 |  | +8 | ^[46]^^[47]^^[48]^ |
| berkelium |  |  |  |  | **Bk** |  | +2 | **+3** | +4 |  |  |  |  | ^[49]^ |
| californium |  |  |  |  | **Cf** |  | +2 | **+3** | +4 |  |  |  |  |  |
| einsteinium |  |  |  |  | **Es** |  | +2 | **+3** | +4 |  |  |  |  | ^[50]^ |
| fermium |  |  |  |  | **Fm** |  | +2 | **+3** |  |  |  |  |  |  |
| mendelevium |  |  |  |  | **Md** |  | +2 | **+3** |  |  |  |  |  |  |
| nobelium |  |  |  |  | **No** |  | **+2** | +3 |  |  |  |  |  |  |
| lawrencium |  |  |  |  | **Lr** |  |  | **+3** |  |  |  |  |  |  |
| rutherfordium |  |  |  |  | **Rf** |  |  |  | **+4** |  |  |  |  |  |
| dubnium |  |  |  |  | **Db** |  |  |  |  | **+5** |  |  |  | ^[51]^ |
| seaborgium |  |  |  |  | **Sg** |  |  |  |  |  | **+6** |  |  | ^[52]^ |
| bohrium |  |  |  |  | **Bh** |  |  |  |  |  |  | **+7** |  | ^[53]^ |
| hassium |  |  |  |  | **Hs** |  |  |  |  |  |  |  | **+8** | ^[54]^ |

A figure with a similar format (shown below) was used by Irving Langmuir in 1919 in one of the early papers about the octet rule.^[55]^ The periodicity of the oxidation states was one of the pieces of evidence that led Langmuir to adopt the rule.

## References and notes[edit]

1. ^ Jump up to: ^***a***^ ^***b***^ Greenwood, Norman N.; Earnshaw, Alan (1997). *Chemistry of the Elements* (2nd ed.). Butterworth-Heinemann. pp. 27–28. ISBN 0080379419.
2. **Jump up ^** Be(I) has been observed in beryllium monohydride (BeH); see "Beryllium: Beryllium(I) Hydride compound data". bernath.uwaterloo.ca. Retrieved 2007-12-10.
3. **Jump up ^** Low valent magnesium compounds with Mg(I) have been obtained using bulky ligands; see Green, S. P.; Jones C.; Stasch A. (December 2007). "Stable Magnesium(I) Compounds with Mg-Mg Bonds". *Science* **318** (5857): 1754–1757. Bibcode:2007Sci...318.1754G. doi:10.1126/science.1150856. PMID 17991827.  Cite uses deprecated parameters (help)
4. **Jump up ^** Al(II) has been observed in aluminium(II) oxide (AlO); see D. C. Tyte (1964). "Red (B2Π–A2σ) Band System of Aluminium Monoxide". *Nature* **202** (4930): 383. Bibcode:1964Natur.202..383T. doi:10.1038/202383a0. , and in dialanes (R_2_Al—AlR_2_);
   see Uhl, Werner "Organoelement Compounds Possessing Al—Al, Ga—Ga,
   In—In, and Tl—Tl Single Bonds" Advances in Organometallic Chemistry
   Volume 51, 2004, Pages 53–108. doi:10.1016/S0065-3055(03)51002-4
5. **Jump up ^** Ca(I) has been observed; see Krieck,
   Sven; Görls, Helmar; Westerhausen, Matthias (2010). "Mechanistic
   Elucidation of the Formation of the Inverse Ca(I) Sandwich Complex
   [(thf)3Ca(μ-C6H3-1,3,5-Ph3)Ca(thf)3] and Stability of Aryl-Substituted
   Phenylcalcium Complexes". *Journal of the American Chemical Society* **132** (35): 12492–501. doi:10.1021/ja105534w. PMID 20718434.
6. **Jump up ^** Ti(I) has been observed in titanium monohydride (TiH); see Andersson, N. *et al.*; Balfour, Walter J.; Bernath, Peter F.; Lindgren, Bo; Ram, Ram S. (2003). "Emission spectra of TiH and TiD near 938 nm". *J. Chem. Phys.* **118** (8): 10543. Bibcode:2003JChPh.118.3543A. doi:10.1063/1.1539848.
7. **Jump up ^** Fe(VII) and Fe(VIII) have been reported; see Yurii
   D. Perfiliev; Virender K. Sharma (2008). "Higher Oxidation States of
   Iron in Solid State: Synthesis and Their Mössbauer Characterization –
   Ferrates – ACS Symposium Series (ACS Publications)". *Platinum Metals Review* **48** (4): 157. doi:10.1595/147106704X10801.  However, their existence has been disputed.
8. **Jump up ^** Zn(I) has been observed in Zn_2_Cl_2_; see Holleman, Arnold F.; Wiberg, Egon; Wiberg, Nils; (1985). "Zink". *Lehrbuch der Anorganischen Chemie* (in German) (91–100 ed.). Walter de Gruyter. pp. 1034–1041. ISBN 3-11-007511-3.  Cite uses deprecated parameters (help)
9. **Jump up ^** Ge(−1), Ge(−2), and Ge(−3) have been observed in germanes; see Greenwood, Norman N.; Earnshaw, Alan (1997). *Chemistry of the Elements* (2nd ed.). Butterworth-Heinemann. ISBN 0080379419. .
10. **Jump up ^** As(I) has been observed in arsenic(I) iodide (AsI); see Ellis,
    Bobby D.; MacDonald, Charles L. B. (2004). "Stabilized Arsenic(I)
    Iodide: A Ready Source of Arsenic Iodide Fragments and a Useful Reagent
    for the Generation of Clusters". *Inorganic Chemistry* **43** (19): 5981–6. doi:10.1021/ic049281s. PMID 15360247.
11. **Jump up ^** Se(I) has been observed in selenium(I) chloride (Se_2_Cl_2_); see "Selenium : Selenium(I) chloride compound data". WebElements.com. Retrieved 2007-12-10.
12. **Jump up ^** Sr(I) has been observed in strontium monofluoride (SrF); see P. Colarusso *et al.*; Guo, B.; Zhang, K.-Q.; Bernath, P.F. (1996). "High-Resolution Infrared Emission Spectrum of Strontium Monofluoride". *J. Molecular Spectroscopy* **175**: 158. Bibcode:1996JMoSp.175..158C. doi:10.1006/jmsp.1996.0019.
13. **Jump up ^** Y(I) has been observed in yttrium(I) bromide (YBr); see "Yttrium: yttrium(I) bromide compound data". OpenMOPAC.net. Retrieved 2007-12-10.
14. **Jump up ^** Y(II) has been observed in yttrium(II) hydride (YH_2_); see "Yttrium: yttrium(II) hydride compound data". WebElements.com. Retrieved 2007-12-10.
15. **Jump up ^** Nb(I) has been observed in niobium monochloride (NbCl); see Ram, R.S.; Rinskopf, N.; Liévin, J.; Bernatha, P.F. (2004). "Fourier transform emission spectroscopy and ab initio calculations on NbCl". *Journal of Molecular Spectroscopy* **228** (2): 544–553. Bibcode:2004JMoSp.228..544R. doi:10.1016/j.jms.2004.02.001.  Cite uses deprecated parameters (help)
16. **Jump up ^** Pd(I) has been observed; see Crabtree, R. H. (2002). "CHEMISTRY: A New Oxidation State for Pd?". *Science* **295** (5553): 288. doi:10.1126/science.1067921.
17. **Jump up ^** Pd(VI) complexes have been observed; see Chen, W.; Shimada, Shigeru; Tanaka, Masato (2002). "Synthesis and Structure of Formally Hexavalent Palladium Complexes". *Science* **295** (5553): 308. Bibcode:2002Sci...295..308C. doi:10.1126/science.1067027.
18. **Jump up ^** Ag(IV) has been observed in potassium hexafluoroargentate (K_2_AgF_6_) and caesium hexafluoroargentate (Cs_2_AgF_6_); see Riedel, Sebastian; Kaupp, Martin (2009). "The highest oxidation states of the transition metal elements". *Coordination Chemistry Reviews* (Elsevier) **253** (5–6): 606–624. doi:10.1016/j.ccr.2008.07.014.
19. **Jump up ^** Cd(I) has been observed in cadmium(I) tetrachloroaluminate (Cd_2_(AlCl_4_)_2_); see Holleman, Arnold F.; Wiberg, Egon; Wiberg, Nils; (1985). "Cadmium". *Lehrbuch der Anorganischen Chemie* (in German) (91–100 ed.). Walter de Gruyter. pp. 1056–1057. ISBN 3-11-007511-3.  Cite uses deprecated parameters (help)
20. **Jump up ^** I(IV) has been observed in iodine dioxide (IO_2_); see Pauling, Linus (1988). "Oxygen Compounds of Nonmetallic Elements". *General Chemistry* (3rd ed.). Dover Publications, Inc. p. 259. ISBN 0-486-65622-5.
21. **Jump up ^** Xe(I) has been observed in xenon hexafluoroplatinate and xenon hexafluororhodate; see Pauling, Linus (1988). *General Chemistry* (3rd ed.). Dover Publications, Inc. p. 250. ISBN 0-486-65622-5.
22. **Jump up ^** Nd(IV) has been observed in unstable solid state compounds; see Holleman, A. F.; Wiberg, E. (2001), *Inorganic Chemistry*, San Diego: Academic Press, ISBN 0-12-352651-5
23. **Jump up ^** Pm(II) has been observed in dilute, solid solutions of promethium dihalides in alkaline earth dihalides; see Holleman, A. F.; Wiberg, E. (2001), *Inorganic Chemistry*, San Diego: Academic Press, ISBN 0-12-352651-5
24. **Jump up ^** Tb(II) has been observed in terbium dihydride (TbH_2_), terbium(II) selenide (TbSe), and terbium(II) telluride (TbTe); see Patnaik, Pradyot (2003). *Handbook of Inorganic Chemical Compounds*. McGraw-Hill. pp. 920–921. ISBN 0-07-049439-8. Retrieved 2009-06-06.
25. **Jump up ^** Dy(IV) has been observed in unstable solid state compounds; see Holleman, A. F.; Wiberg, E. (2001), *Inorganic Chemistry*, San Diego: Academic Press, ISBN 0-12-352651-5
26. **Jump up ^** Ho(II) has been observed in dilute, solid solutions of holmium dihalides in alkaline earth dihalides; see Holleman, A. F.; Wiberg, E. (2001), *Inorganic Chemistry*, San Diego: Academic Press, ISBN 0-12-352651-5
27. **Jump up ^** Er(II) has been observed in dilute, solid solutions of erbium dihalides in alkaline earth dihalides; see Holleman, A. F.; Wiberg, E. (2001), *Inorganic Chemistry*, San Diego: Academic Press, ISBN 0-12-352651-5
28. **Jump up ^** Os(−1) has been observed in Na  
    2[Os  
    4(CO)  
    13]; see Krause, J.;
    Siriwardane, Upali; Salupo, Terese A.; Wermer, Joseph R.; Knoeppel,
    David W.; Shore, Sheldon G. (1993). "Preparation of [Os_3_(CO)_11_]^2−^ and its reactions with Os_3_(CO)_12_; structures of [Et_4_N] [HOs_3_(CO)_11_] and H_2_OsS_4_(CO)". *Journal of Organometallic Chemistry* **454**: 263–271. doi:10.1016/0022-328X(93)83250-Y.  and Carter,
    Willie J.; Kelland, John W.; Okrasinski, Stanley J.; Warner, Keith E.;
    Norton, Jack R. (1982). "Mononuclear hydrido alkyl carbonyl complexes of
    osmium and their polynuclear derivatives". *Inorganic Chemistry* **21** (11): 3955–3960. doi:10.1021/ic00141a019.  Cite uses deprecated parameters (help)
29. **Jump up ^** Ir(−3) has been observed in Ir(CO)_3_^3−^; see Greenwood, Norman N.; Earnshaw, Alan (1997). *Chemistry of the Elements* (2nd ed.). Butterworth-Heinemann. p. 1117. ISBN 0080379419.
30. **Jump up ^** Ir(VII) has been observed in iridium heptafluoride (IrF_7_); see Prediction of new compounds and new oxidation states.
31. **Jump up ^** Ir(VIII) has been observed in iridium tetroxide (IrO_4_); see Gong,
    Yu; Zhou, Mingfei; Kaupp, Martin; Riedel, Sebastian (2009). "Formation
    and Characterization of the Iridium Tetroxide Molecule with Iridium in
    the Oxidation State +VIII". *Angewandte Chemie International Edition* **48** (42): 7879. doi:10.1002/anie.200902733.
32. **Jump up ^** Pt(−1) and Pt(−2) have been observed in the barium platinides Ba_2_Pt, BaPt, and Ba_3_Pt_2_ respectively: see Karpov,
    Andrey; Konuma, Mitsuharu; Jansen, Martin (2006). "An experimental
    proof for negative oxidation states of platinum: ESCA-measurements on
    barium platinides". *Chemical Communications* (8): 838–840. doi:10.1039/b514631c. PMID 16479284.
33. **Jump up ^** Pt(I) and Pt(III) have been observed in bimetallic and polymetallic species; see Kauffman, George B.; Thurner, Joseph J.; Zatko, David A. (1967). "Ammonium Hexachloroplatinate(IV)". *Inorganic Syntheses*. Inorganic Syntheses **9**: 182–185. doi:10.1002/9780470132401.ch51. ISBN 978-0-470-13240-1.
34. **Jump up ^** Hg(IV) has been observed in mercury(IV) fluoride (HgF_4_); see Xuefang
    Wang; Lester Andrews; Sebastian Riedel; and Martin Kaupp (2007).
    "Mercury Is a Transition Metal: The First Experimental Evidence for HgF_4_". *Angew. Chem. Int. Ed.* **46** (44): 8371–8375. doi:10.1002/anie.200703710. PMID 17899620.  Cite uses deprecated parameters (help).
35. **Jump up ^** Tl(−1) has been observed in caesium thallide
    (CsTl); see Bashilova NI & Khomutova, TV 1984, 'Thallates of alkali
    metals and monovalent thallium formed in aqueous solutions of their
    hydroxides', *Russian Chemical Bulletin,* vol. 33, no. 8, August, pp. 1543–47.
36. **Jump up ^** Bi(I) has been observed in bismuth monobromide (BiBr) and bismuth monoiodide (BiI); see Godfrey, S. M.; McAuliffe, C. A.; Mackie, A. G.; Pritchard, R. G. (1998). Nicholas C. Norman, ed. *Chemistry of arsenic, antimony, and bismuth*. Springer. pp. 67–84. ISBN 0-7514-0389-X.
37. **Jump up ^** Po(V) has been observed in dioxidopolonium(1+) (PoO+  
    2); see Thayer, John S. (2010). *Chemistry of heavier main group elements*. p. 78. doi:10.1007/9781402099755\_2.
38. **Jump up ^** Rn(II) has been observed in radon difluoride (RnF_2_); see Stein, L. (1970). "Ionic Radon Solution". *Science* **168** (3929): 362–4. Bibcode:1970Sci...168..362S. doi:10.1126/science.168.3929.362. PMID 17809133.  and Kenneth S. Pitzer (1975). "Fluorides of radon and element 118". *J. Chem. Soc., Chem. Commun.,* (18): 760b – 761. doi:10.1039/C3975000760b.
39. **Jump up ^** Rn(IV) is reported by Greenwood and Earnshaw, but is not known to exist; see Sykes, A. G. (1998). "Recent Advances in Noble-Gas Chemistry". *Advances in Inorganic Chemistry* **46**. Academic Press. pp. 91–93. ISBN 978-0-12-023646-6. Retrieved 22 November 2012.
40. **Jump up ^** Rn(VI) is known in radon trioxide (RnO_3_); see Sykes, A. G. (1998). "Recent Advances in Noble-Gas Chemistry". *Advances in Inorganic Chemistry* **46**. Academic Press. pp. 91–93. ISBN 978-0-12-023646-6. Retrieved 22 November 2012.
41. **Jump up ^** Ac(II) is known in actinium dihydride (AcH_2_); see Farr, J; Giorgi, A.L.; Bowman, M.G.; Money, R.K. (1961). "The crystal structure of actinium metal and actinium hydride". *Journal of Inorganic and Nuclear Chemistry* **18**: 42. doi:10.1016/0022-1902(61)80369-2.
42. **Jump up ^** Pa(II) is known in protactinium(II) oxide (PaO); see Sellers,
    Philip A.; Fried, Sherman; Elson, Robert E.; Zachariasen, W. H. (1954).
    "The Preparation of Some Protactinium Compounds and the Metal". *Journal of the American Chemical Society* **76** (23): 5935. doi:10.1021/ja01652a011.
43. **Jump up ^** U(II) has been observed in uranium monoxide (UO), uranium monosulfide (US), uranium acetylide (UC_2_), and uranium sesquicarbide (U_2_C_3_); see A.L. Bowman, G.P. Arnold, W.G. Witteman, T.C. Wallace and N.G. Nereson, Acta Crystallographica, 1966, **21**, 670–671.
44. **Jump up ^** Unstable Pu(VIII) complexes can form in alkaline solutions; see Kiselev, Yu. M.; Nikonov, M. V.; Tananaev, I. G.; Myasoedov, B. F. (2009). "On the Existence of Plutonium Tetroxide". *Doklady Akademii Nauk* (Pleiades Publishing, Ltd.) **425** (5): 634–637. doi:10.1134/S0012501609040022. ISSN 0012-5016. Retrieved February 25, 2012.
45. **Jump up ^** Am(VII) has been observed in AmO5−  
    6; see Americium,
    Das Periodensystem der Elemente für den Schulgebrauch (The periodic
    table of elements for schools) chemie-master.de (in German), Retrieved
    28 November 2010 and Greenwood, Norman N.; Earnshaw, Alan (1997). *Chemistry of the Elements* (2nd ed.). Butterworth-Heinemann. p. 1265. ISBN 0080379419.
46. **Jump up ^** Cm(II) has been observed in curium(II) oxide (CmO); see Holleman, Arnold F. and Wiberg, Nils *Textbook of Inorganic Chemistry*, p.1972, 102 Edition, de Gruyter, Berlin 2007, ISBN 978-3-11-017770-1.
47. **Jump up ^** Cm(VI) has been observed in curium trioxide (CmO_3_) and dioxidocurium(2+) (CmO2+  
    2); see Domanov, V. P.; Lobanov, Yu. V. (October 2011). "Formation of volatile curium(VI) trioxide CmO_3_". *Radiochemistry* (SP MAIK Nauka/Interperiodica) **53** (5): 453–6. doi:10.1134/S1066362211050018.
48. **Jump up ^** Cm(VIII) has been observed in curium tetroxide (CmO_4_); see Domanov,
    V. P. (January 2013). "Possibility of generation of octavalent curium
    in the gas phase in the form of volatile tetraoxide CmO_4_". *Radiochemistry* (SP MAIK Nauka/Interperiodica) **55** (1): 46–51. doi:10.1134/S1066362213010098.
49. **Jump up ^** Bk(II) has been observed in berkelium(II) oxide (BkO); see Peterson J. R. and Hobart D. E. "The Chemistry of Berkelium" in Harry Julius Emeléus (Ed.) *Advances in inorganic chemistry and radiochemistry*, Volume 28, Academic Press, 1984 ISBN 0-12-023628-1, p. 51, doi:10.1016/S0898-8838(08)60204-4
50. **Jump up ^** Es(IV) is known in einsteinium(IV) fluoride (EsF_4_); see Kleinschmidt, P (1994). "Thermochemistry of the actinides". *Journal of Alloys and Compounds*. 213–214: 169. doi:10.1016/0925-8388(94)90898-2.
51. **Jump up ^** Db(V) has been observed in dubnium pentachloride (DbCl_5_); see H. W. Gäggeler (2007). "Gas Phase Chemistry of Superheavy Elements". Paul Scherrer Institute. pp. 26–28.
52. **Jump up ^** Sg(VI) has been observed in seaborgium oxide hydroxide (SgO_2_(OH)_2_); see Huebener et al.; Taut, S.; Vahle, A.; Dressler, R.; Eichler, B.; Gäggeler, H. W.; Jost, D.T.; Piguet, D. et al. (2001). "Physico-chemical characterization of seaborgium as oxide hydroxide". *Radiochim. Acta* **89** (11–12\_2001): 737–741. doi:10.1524/ract.2001.89.11-12.737.
53. **Jump up ^** Bh(VII) has been observed in bohrium oxychloride (BhO_3_Cl); see "Gas chemical investigation of bohrium (Bh, element 107)", Eichler et al., *GSI Annual Report 2000*. Retrieved on 2008-02-29
54. **Jump up ^** Hs(VIII) has been observed in hassium tetroxide (HsO_4_); see "Chemistry of Hassium" (PDF). *Gesellschaft für Schwerionenforschung mbH*. 2002. Retrieved 2007-01-31.
55. **Jump up ^** Langmuir, Irving (1919). "The arrangement of electrons in atoms and molecules". *J. Am. Chem. Soc.* **41** (6): 868–934. doi:10.1021/ja02227a002.

## See also[edit]

| [hide] - v - t - e  Chemical elements data | |
| --- | --- |
|  | |
| Elements | - List of elements — atomic mass, atomic number, symbol, name - Periodic table |
|  | |
| Data | - Abundance of the chemical elements in Earth's crust, sea water, Sun and solar system   - data page  - Atomic radius empirical, calculated, van der Waals radius, covalent radius   - data page  - Boiling point   - data page  - Critical point   - data page  - Density solid, liquid, gas   - data page  - Elastic properties of the elements: Young's modulus, Poisson's ratio, bulk modulus, shear modulus   - data page  - Electrical resistivity   - data page  - Electron affinity   - data page  - Electron configuration   - data page  - Electronegativity (Pauling scale)   - data page  - Hardness: Mohs hardness, Vickers hardness, Brinell hardness   - data page  - Heat capacity   - data page  - Heat of fusion   - data page  - Heat of vaporization   - data page  - Ionization energy (in eV) and molar ionization energies (in kJ/mol)   - data page  - Melting point   - data page  - Molar ionization energy  - Oxidation state   - **data page**  - Speed of sound   - data page  - Thermal conductivity   - data page  - Thermal expansion   - data page  - Vapor pressure   - data page |

| [show] - v - t - e  Periodic table | |
| --- | --- |
|  | |
| Standard form | - Periodic table (18 columns) - With large cells   - 18 columns wide   - 32 columns wide |
|  | |
| Extended form | - Extension beyond the 7th period   - Fricke model   - Pyykkö model   - Extended form, large cells |
|  | |
| Alternative structure | - Alternatives - Chemical Galaxy - Janet's Left Step table |
|  | |
| Groups | - 1 (Alkali metals) - 2 (Alkaline earth metals) - 3 - 4 - 5 - 6 - 7 - 8 - 9 - 10 - 11 - 12 - 13 (Boron group) - 14 (Carbon group) - 15 (Pnictogens) - 16 (Chalcogens) - 17 (Halogens) - 18 (Noble gases) |
|  | |
| Periods | - 1 - 2 - 3 - 4 - 5 - 6 - 7 - 8 - 9 |
|  | |
| Blocks | - s-block - p-block - d-block - f-block - g-block |
|  | |
| Metals–nonmetals | - Metals - Metalloids   - dividing line - Nonmetals - Subgroups: Alkali metals - Alkaline earth metals - Lanthanides - Actinides - Superactinides - Eka-superactinides - Transition metals - Other metals - Metalloids - Polyatomic nonmetals - Diatomic nonmetals - Noble gases |
|  | |
| History | - History of the periodic table - Element discoveries   - Mendeleev's predictions - Name etymology   - controversies   - places   - scientists |
|  | |
| Element properties | - Atomic weights - Crystal structure - Dietary elements - Electron affinities - Electron configurations - Electronegativity by Allen scale - Electronegativity by Pauling scale - Goldschmidt classification - Maximum valences - Nutritional elements - Platinum group metals (PGM) - Rare earth elements - Refractory metals |
|  | |
| List of elements by | - Abundance (in humans) - Atomic properties - Nuclear stability - Production - Symbol |
|  | |
| Data pages | - Abundances - Atomic radii - Boiling points - Critical points - Densities - Elastic properties - Electrical resistivities - Electron affinities - Electron configurations - Electronegativities - Hardnesses - Heat capacities - Heats of fusion - Heats of vaporization - Ionization energies - Melting points - **Oxidation states** - Speeds of sound - Thermal conductivities - Thermal expansion coefficients - Vapor pressures |
|  | |
| See also | - Names for sets of chemical elements - IUPAC   - nomenclature   - systematic element name - Trivial name - Dmitri Mendeleev - Periodic table (simplified Chinese) - Periodic table (traditional Chinese) |
|  | |
| - **Book** - **Category** - **Chemistry Portal** | |

Retrieved from "http://en.wikipedia.org/w/index.php?title=List\_of\_oxidation\_states\_of\_the\_elements&oldid=606539669"

Categories:

- Lists of chemical elements

Hidden categories:

- Pages containing cite templates with deprecated parameters

## Navigation menu

### Personal tools

- Create account
- Log in

### Namespaces

- Article
- Talk

### Variants

### Views

- Read
- Edit
- View history

### Actions

### Search

### Navigation

- Main page
- Contents
- Featured content
- Current events
- Random article
- Donate to Wikipedia
- Wikimedia Shop

### Interaction

- Help
- About Wikipedia
- Community portal
- Recent changes
- Contact page

### Tools

- What links here
- Related changes
- Upload file
- Special pages
- Permanent link
- Page information
- Data item
- Cite this page

### Print/export

- Create a book
- Download as PDF
- Printable version

### Languages

- العربية
- Bosanski
- Deutsch
- Ελληνικά
- Español
- فارسی
- Italiano
- Polski
- Português
- Русский
- Suomi
- ไทย
- 中文

Edit links

- This page was last modified on 30 April 2014 at 21:54.
- Text is available under the Creative Commons Attribution-ShareAlike License;
  additional terms may apply. By using this site, you agree to the Terms of Use and Privacy Policy. Wikipedia® is a registered trademark of the Wikimedia Foundation, Inc., a non-profit organization.

- Privacy policy
- About Wikipedia
- Disclaimers
- Contact Wikipedia
- Developers
- Mobile view
