# Supplementary material for: COD::CIF::Parser: an error-correcting CIF parser for the Perl language
Source: J Appl Crystallogr. 2016 Feb 1;49(Pt 1):292–301. doi: 10.1107/S1600576715022396 (PMC4762566; doi:10.1107/S1600576715022396)
Supplement: Supplementary file 3 [file j-49-00292-sup3.tgz › cod-tools-1.0/doc/database-scheme-draft.pdf]

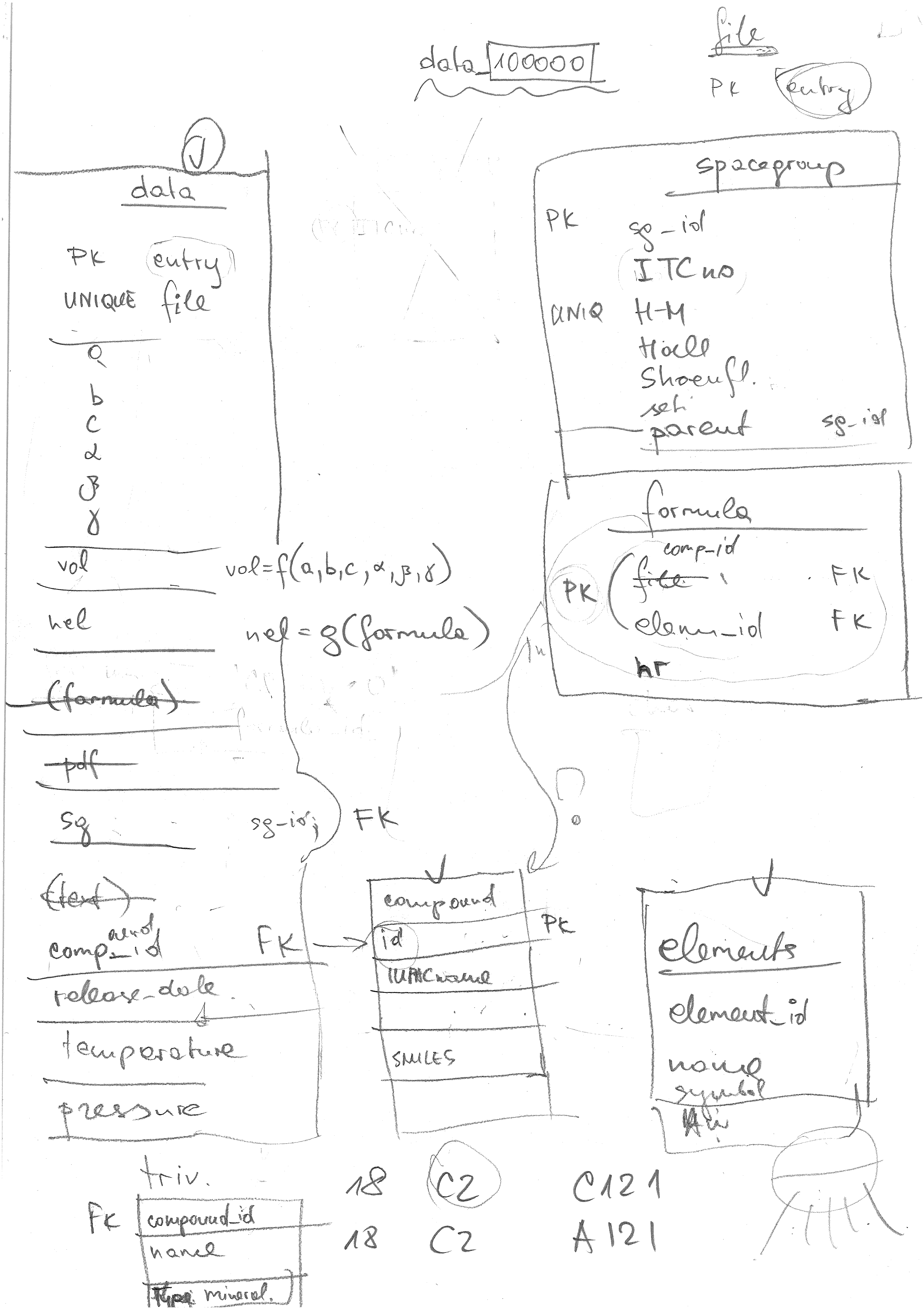
file
PK entry
data_100000
data
PK entry
UNIQUE file
a
b
c
α
β
γ
vol
vol=f(a,b,c,α,β,γ)
hel
hel=g(formula)
(formula)
pdf
sg
sg-id FK
(text)
compound_id FK
release_date
temperature
pressure
spacegroup
PK sg_id
ITC no
UNIQ H-M
Hall
Schoenfl.
set
parent sg-id
formula
comp_id
PK file FK
element_id FK
nr
compound
id PK
IUPAC name
SMILES
elements
element_id
name
symbol
triv.
FK compound_id
name
Type mineral.
18 C2 C121
18 C2 A121

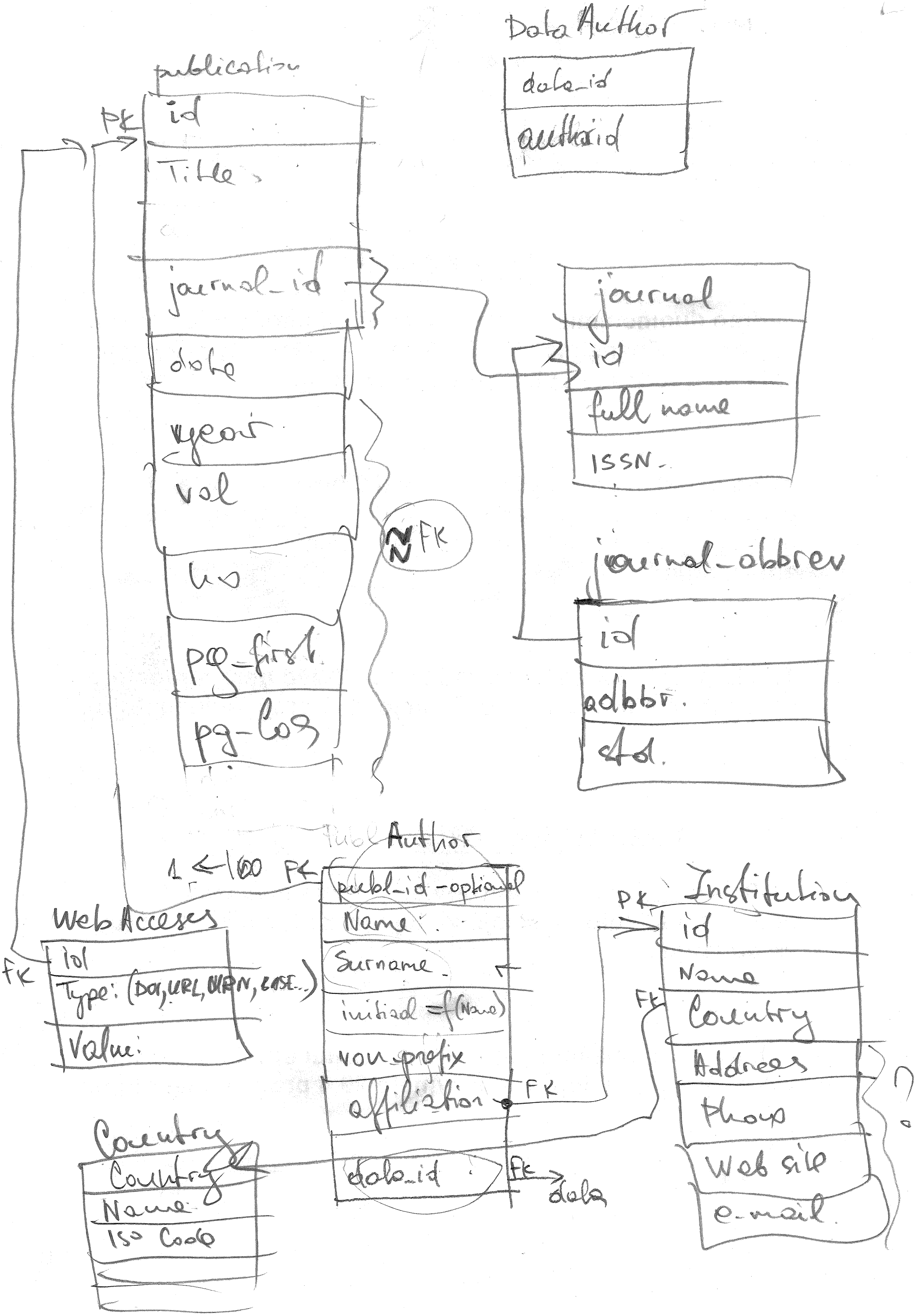
DataAuthor
data_id
authorid
publication
PK
id
Title
journal_id
date
year
vol
no
pg_first
pg_last
FK
journal
id
full name
ISSN.
journal_abbrev
id
abbr.
std.
Publ Author
1 ≤ 100
PK
publ_id -optional
Name
Surname
initial = f(Name)
von_prefix
affiliation
FK
data_id
Fk → data
Institution
PK
id
Name
FK
Country
Address
Phone
Web site
e-mail
?
WebAcceses
FK
id
Type: (DOI, URL, URN, EASE...)
Value:
Country
Country
Name
ISO Code

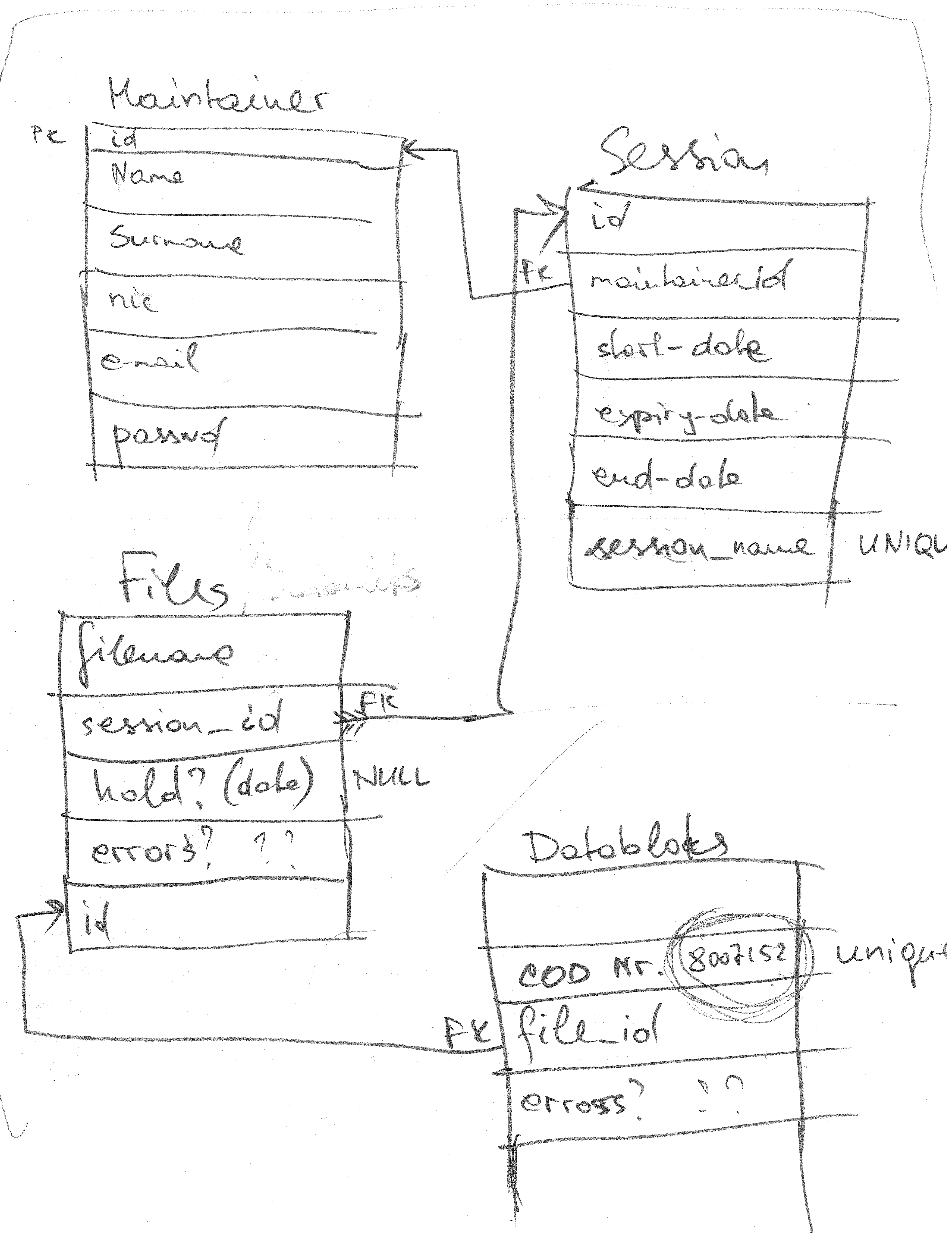
Maintainer
PK
id
Name
Surname
nic
e-mail
passwd
Session
id
FK
maintainer_id
start-date
expiry-date
end-date
session_name
UNIQU
Files
filename
session_id
FK
hold? (date)
NULL
errors? ??
id
Datablocks
COD Nr.
8007152
unique
FK
file_id
erross? ??

Alter.

<u>data</u>

~~entry PK/UNIQUE~~

file UNIQUE

a

b

c

α

β

γ

vol

nele

→ formula 'C2uH6uO'

title ' '

Journal

year

volume

issue

firstpg

lastpg

→ Authors 'F. Last; L, F; F von L'

trivnames 'glycerol;glycerin'

iupac

mineral

smiles

molwt

Title

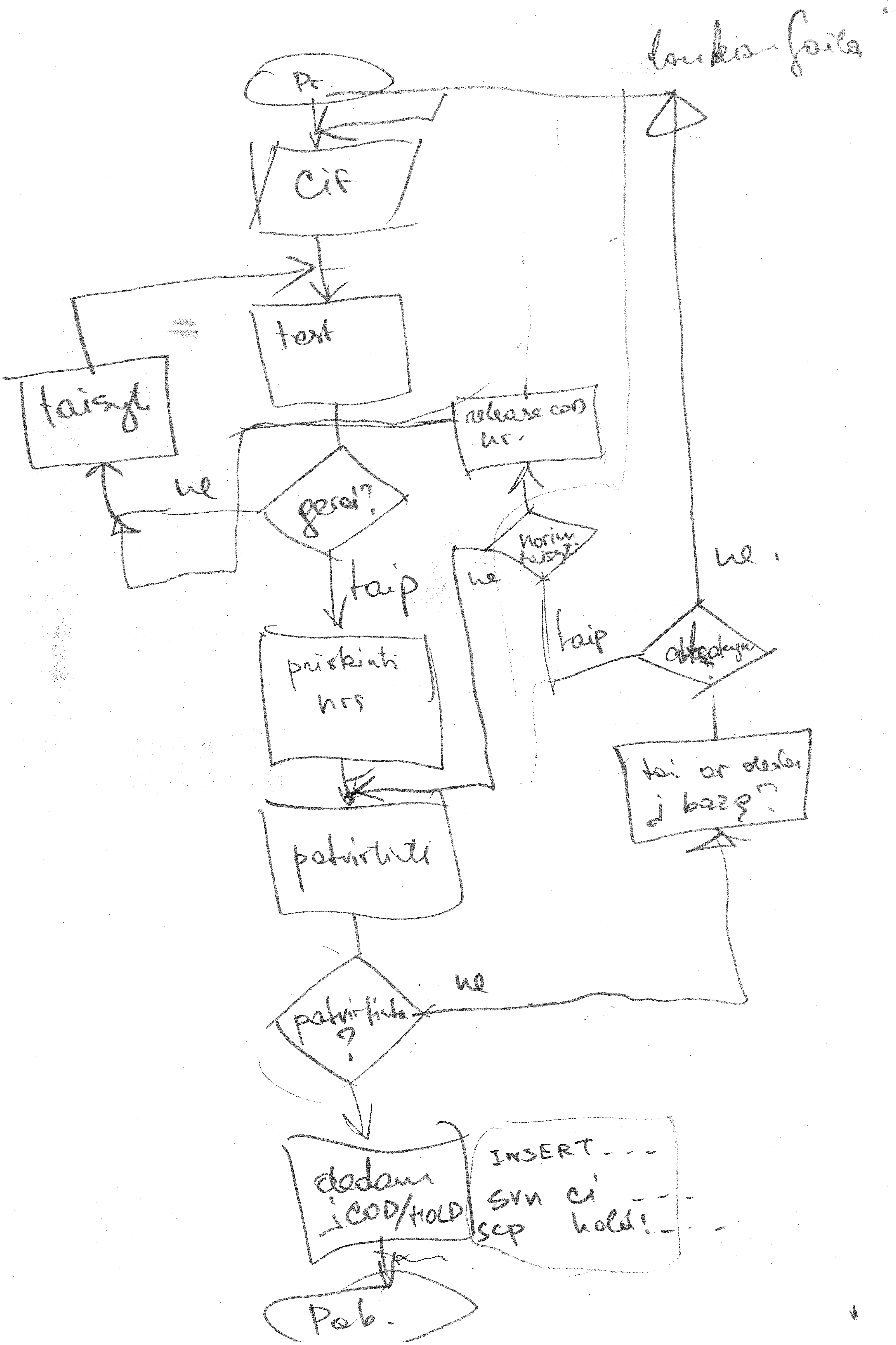
Pr.
cif
test
taisyt.
gerai?
ne
taip
priskinti nrs
patvirtinti
patvirtinta ?
ne
release con nr.
ne
taip
ne
dedam į COD/HOLD
INSERT . . .
svn ci . . .
scp hold! . .
Pab.

1) Autorius ← !

2) Affiliation ← !

Yu.

StructAuthors

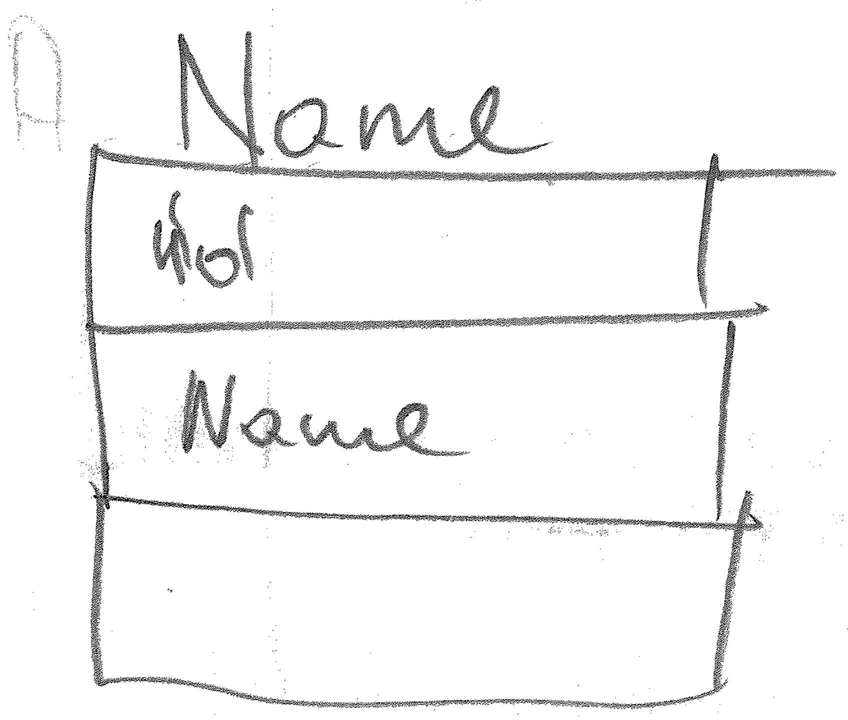

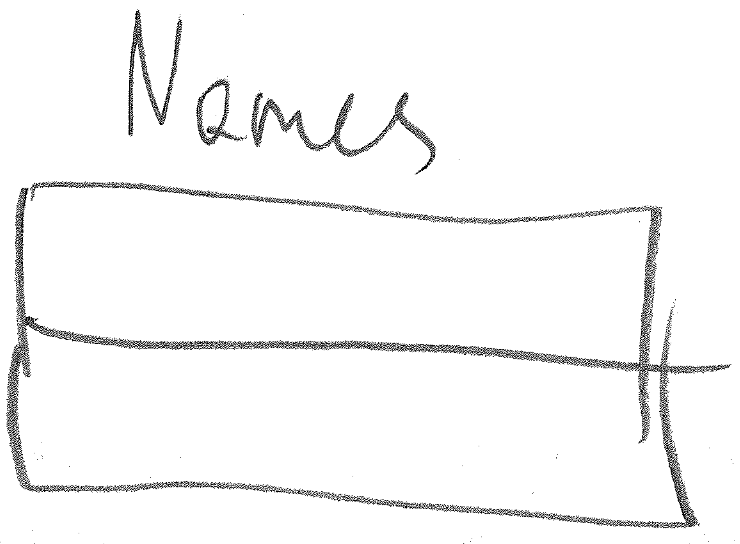

CIF

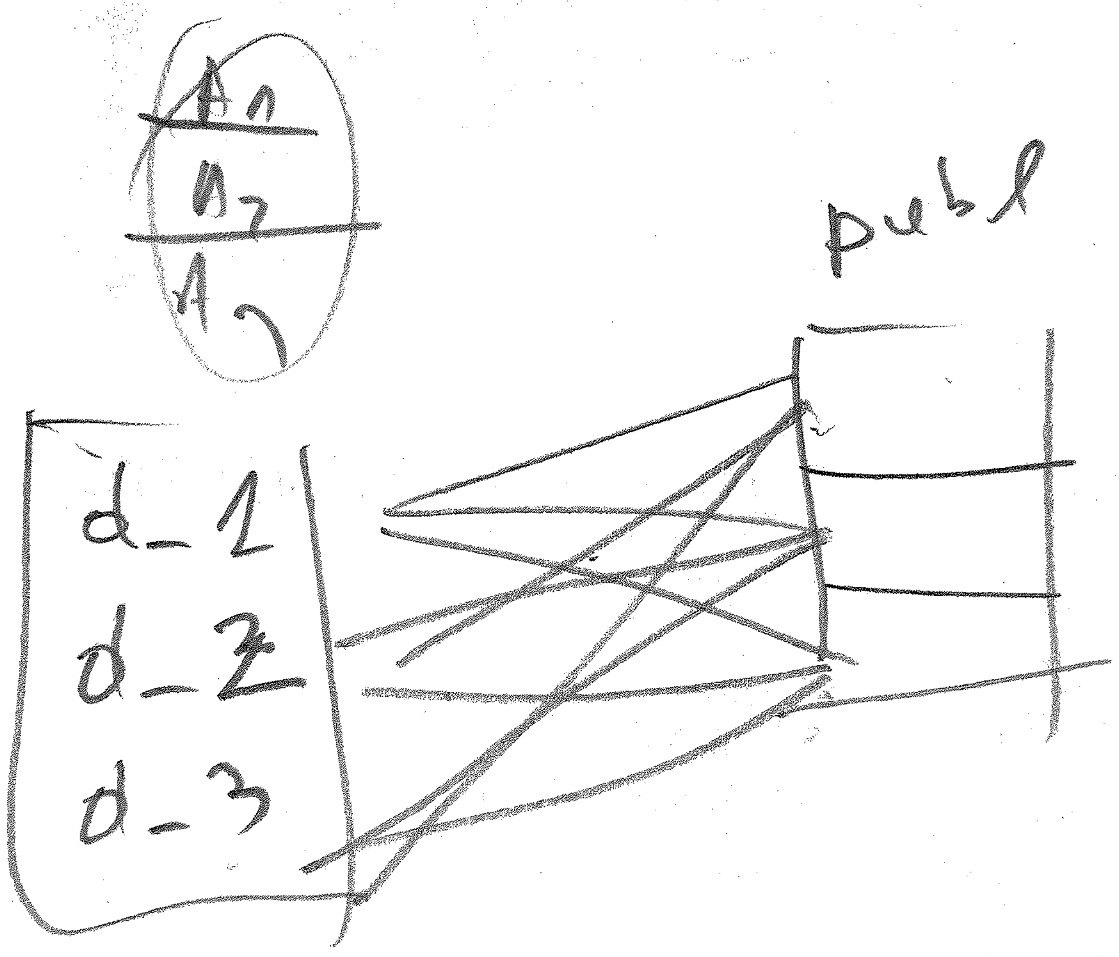

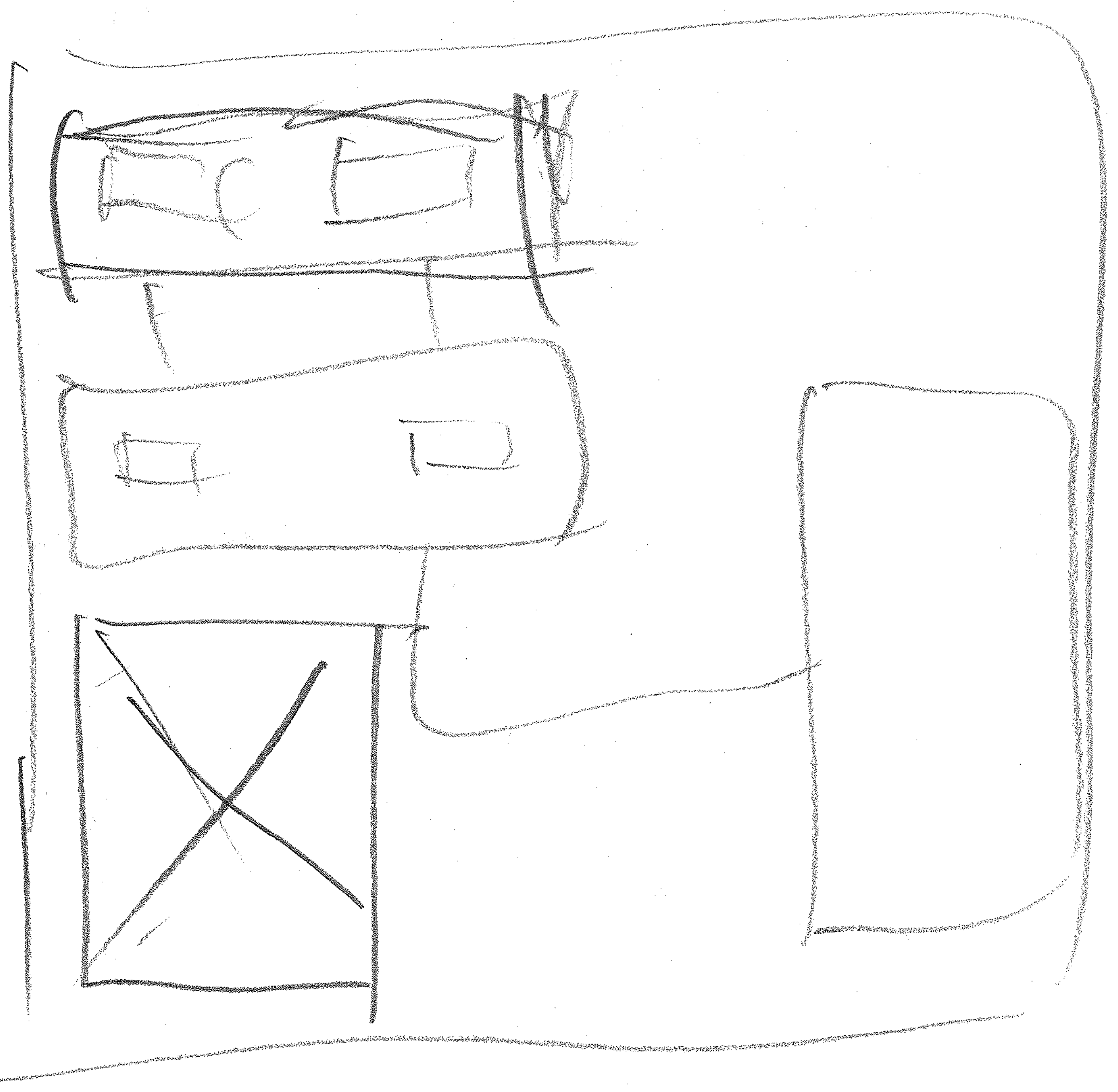

i18n

~~l16n~~

l10n

SAULIUS

GRAZULIS

GRA8LIS
